# 3D Face Reconstruction with Deep Learning: Architectures, Datasets, and Benchmark Analysis

**DOI:** 10.3390/s26082540

**Published:** 2026-04-20

**Authors:** Sankarshan Dasgupta, Ju Shen, Tam V. Nguyen

**Affiliations:** Department of Computer Science, University of Dayton, Dayton, OH 45469, USA; jshen1@udayton.edu (J.S.); tnguyen1@udayton.edu (T.V.N.)

**Keywords:** 3D face reconstruction, monocular RGB, sensor-aware systems, camera calibration, benchmark evaluation

## Abstract

Three-Dimensional (3D) face reconstruction from monocular Red-Green-Blue (RGB) imagery remains a fundamental yet ill-posed challenge in computer vision, with applications in biometrics, augmented reality/virtual reality (AR/VR), and intelligent visual sensing systems. While deep learning has significantly improved reconstruction fidelity and realism, existing surveys primarily focus on network architectures in isolation, often overlooking how sensing conditions, data acquisition protocols, and geometric calibration influence reconstruction reliability and evaluation outcomes. This paper presents a sensor-aware, end-to-end review of deep learning-based 3D face reconstruction and introduces a unified modular framework that connects sensing hardware, data acquisition, calibration, representation learning, and geometric refinement within a coherent pipeline. The reconstruction process is organized into four stages: sensor-driven acquisition and calibration, landmark estimation and feature extraction, 3D representation and parameter regression, and iterative refinement via differentiable rendering. Within this framework, we examine how sensor characteristics, calibration accuracy, representation models, and supervision strategies affect reconstruction accuracy, perceptual quality, robustness, and computational efficiency. We further synthesize the reported results across widely used benchmarks using both geometric and perceptual metrics, highlighting trade-offs between reconstruction fidelity and deployment constraints. By integrating sensing-aware analysis with architectural evaluation, this survey provides practical insights for developing scalable and reliable 3D face reconstruction systems under real-world conditions.

## 1. Introduction

Reconstructing 3D facial geometry from RGB images remains a fundamental challenge in computer vision due to its inherently ill-posed nature [1] and sensitivity to variations in pose, illumination, expression, and occlusion [2,3]. Accurate recovery of 3D facial structure from a single image is critical for applications such as augmented reality, animation, and biometrics; however, it remains challenging despite significant advances in deep learning. To mitigate this ambiguity, most modern approaches adopt parametric representations such as 3D morphable models (3DMMs) [4]. When integrated with deep neural networks, these models enable regression-based frameworks that estimate shape and expression parameters directly from 2D inputs [2,3,5,6,7].

Recent developments extend beyond parametric regression by incorporating dense correspondence learning [8], differentiable rendering [9,10], and neural refinement strategies to improve both geometric accuracy and visual realism. These advances introduce trade-offs between reconstruction fidelity and computational efficiency, with high-fidelity models relying on complex supervision and refinement [11,12], while lightweight architectures support real-time inference under resource constraints [13,14]. The effectiveness of these approaches depends not only on model design but also on training data and supervision strategies. While early methods relied on limited 3D scan datasets such as FaceWarehouse [15] and LSFM [16], recent work increasingly leverages synthetic data and weak supervision [17,18] to improve generalization under real-world conditions.

Despite these advances, existing surveys largely focus on architectural developments [19,20] and algorithmic categorization [21], often placing limited emphasis on sensing conditions, acquisition protocols, and geometric calibration. In practice, however, these factors directly influence reconstruction accuracy, robustness, and the reliability of benchmark evaluations. Variations in sensor modality, acquisition pipelines, and calibration fidelity can introduce significant discrepancies in reported performance. These differences make consistent comparison across datasets challenging. To address this gap, this survey adopts a system-level perspective that integrates sensing, data acquisition, representation learning, and evaluation within a unified reconstruction pipeline. Rather than focusing solely on network architectures, we analyze how sensor characteristics, dataset construction, supervision strategies, and evaluation protocols jointly shape reconstruction performance. This perspective provides a more comprehensive and practically relevant understanding of modern 3D face reconstruction systems, to improve generalization and robustness under real-world conditions.

### 1.1. Existing Surveys

Earlier surveys such as that of Zollhöfer et al. [22] provided a comprehensive review of monocular 3D face reconstruction, tracking, and rendering, documenting the evolution of reconstruction techniques over time. Sharma and Kumar [19] present an overview of deep learning-based approaches; however, several architectural categories discussed in their work have since evolved or merged with newer learning paradigms. Kammoun et al. [20] primarily focus on GAN-based methods for 3D face generation but do not provide a unified framework describing how different reconstruction components interact within practical systems. Recent surveys such as that of Zhalgas et al. [23] focus largely on face recognition rather than geometric reconstruction and therefore do not address reconstruction pipelines, sensing configurations, or calibration considerations.

In contrast, the present survey adopts a sensor-aware and systems-oriented perspective that organizes state-of-the-art methods according to architectural design, training strategies, sensing conditions, and evaluation protocols. Our focus is on monocular in-the-wild 2D-to-3D face reconstruction while also discussing extensions to multi-image settings and synthetic data. The survey emphasizes developments from the past decade, with particular attention to how acquisition conditions and hardware constraints influence reconstruction performance.

Table 1 summarizes the coverage of representative surveys across key dimensions, including methodological scope, dataset utilization, sensing considerations, and calibration aspects. Although these works provide valuable overviews of the research landscape, they do not fully capture recent developments such as implicit representations, differentiable rendering, and neural rendering pipelines.

### 1.2. Distinctiveness of the Present Survey

Building on the comparison above, recent surveys primarily emphasize algorithmic categorization and dataset descriptions, reflecting the rapid evolution of deep learning and neural rendering approaches. However, comparatively limited attention has been given to sensor-aware reconstruction and unified end-to-end pipeline analysis. Table 2 further highlights the distinctive contribution of the present survey.

Our survey not only reviews algorithmic progress but also examines how acquisition conditions and camera geometry influence reconstruction fidelity, robustness, and benchmark interpretation. This systems-oriented perspective provides a more practical and implementation-relevant understanding of modern 3D face reconstruction pipelines.

### 1.3. Key Contributions

To bridge the gap between architectural surveys and deployment-oriented analysis, this work makes the following contributions:

- We introduce a sensor-aware end-to-end reconstruction framework that explicitly connects sensing hardware, acquisition protocols, calibration, representation learning, and geometric refinement. This unified formulation enables systematic analysis of how design choices propagate through the reconstruction pipeline.
- We provide a comprehensive implementation-oriented analysis of datasets, sensing modalities, calibration parameters, landmark strategies, and loss formulations, highlighting their impact on reconstruction accuracy, robustness, and generalization.
- We present a cross-paradigm benchmark synthesis spanning parametric regression, direct geometry estimation, and neural rendering methods and analyze geometric fidelity, perceptual quality, and computational efficiency.

### 1.4. Method Taxonomy and Terminology

This taxonomy unifies commonly used but often inconsistently defined categories across prior works, enabling clearer comparison between representation paradigms and evaluation strategies.

- **Parametric Reconstruction:** Methods that regress parameters of statistical facial models such as 3D morphable models (3DMMs). These approaches typically employ landmark-based geometric constraints and differentiable rendering for photometric supervision.
- **Direct Geometry Estimation:** Approaches that directly predict dense facial geometry without relying on predefined parametric bases. These methods operate on position maps, mesh vertices, or UV-space correspondences and frequently utilize vertex-level supervision.
- **Neural Rendering Methods:** Techniques that represent facial geometry and appearance through neural implicit functions or learned radiance fields. These approaches prioritize photorealistic view synthesis and often employ volumetric or implicit rendering strategies.

These categories are used consistently throughout the manuscript to organize method comparisons and benchmark analysis.

### 1.5. Paper Organization

The remainder of this paper is organized according to the key stages of a typical 3D face reconstruction pipeline:

- Section 2 provides an overview of the reconstruction pipeline and introduces commonly used datasets, including depth-based and landmark-based benchmarks.
- Section 2.2, Section 2.3 and Section 2.4 present core components of the pipeline, including facial landmark detection, feature extraction, and 3D face representation models.
- Section 2.5, Section 2.6, Section 2.7 and Section 2.8 describe the main reconstruction stages, including pose estimation, primary 3D reconstruction, geometric refinement, and evaluation metrics.
- Section 3 presents a quantitative benchmark analysis and comparative evaluation of representative state-of-the-art methods.
- Section 4 concludes the survey and discusses open challenges and future research directions.

Overall, the paper is organized to reflect the main stages of modern 3D face reconstruction pipelines, progressing from representation to reconstruction methodologies and evaluation.

## 2. Methodology Overview

**Figure 1:**
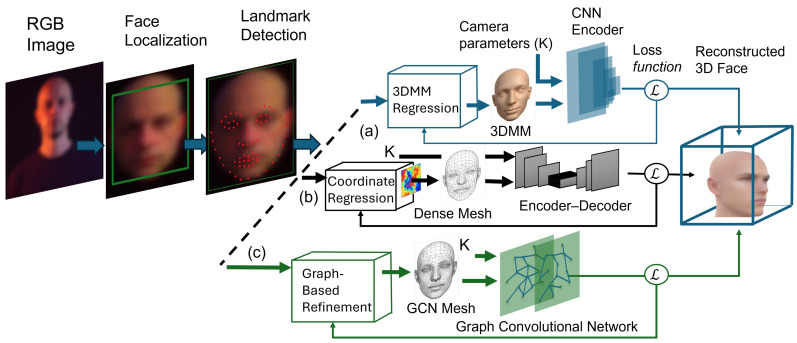
A Modular Reconstruction Pipeline for 3D Face Reconstruction from a Monocular RGB Image. The framework flows from RGB input through face localization and landmark detection to three alternative primary reconstruction strategies: (**a**) parametric regression using linear identity and expression bases with camera parameter estimation and differentiable projection [3]; (**b**) dense coordinate regression via position maps for direct mesh reconstruction [8]; and (**c**) graph-based mesh refinement using graph convolutional networks to capture high-frequency facial details [25]. self-supervised feedback loops optimize the reconstruction using landmark, photometric, perceptual, and geometric loss functions [26]. Not all branches are used simultaneously; they represent alternative approaches reported in the literature. Face localization: green bounding boxes; Red dots: extracted facial landmarks. Dotted lines: alternative reconstruction branches can be selected; Different colored wireframes: distinct reconstruction strategies.

This section introduces a modular implementation framework that organizes diverse deep learning-based methods into a unified 3D face reconstruction pipeline. As illustrated in Figure 1 below, the proposed framework consists of preprocessing, landmark detection, representation modeling, and refinement stages. By adopting a modular perspective, the survey enables researchers and practitioners to understand, compare, and adapt existing approaches according to specific application requirements and available RGB imagery.

### 2.1. Overview of the 3D Face Reconstruction Pipeline

The objective of the 3D face reconstruction pipeline is to recover high-fidelity facial geometry from a monocular RGB image. Due to the inherent ambiguity of inferring three-dimensional structure from two-dimensional observations, the reconstruction process is typically decomposed into several interconnected stages that progressively bridge 2D image measurements and 3D spatial representations. The major stages of the pipeline are summarized below.

- **Data Acquisition**: Collection of facial imagery and associated annotations from datasets used for model training and evaluation.
- **Preprocessing**: Face detection, alignment, cropping, and image normalization to isolate the facial region and reduce variations in scale and illumination.
- **Facial Landmark Detection**: Localization of key facial landmarks that provide geometric constraints for subsequent reconstruction and alignment.
- **3D Face Representation**: Selection of an appropriate representation model (e.g., parametric reconstruction, mesh-based representations, or implicit neural representations) to encode facial shape and appearance.
- **Pose and Camera Modeling**: Estimation of head pose and camera parameters to establish the geometric relationship between the image plane and the 3D facial model.
- **Primary Reconstruction**: Regression of model parameters or direct prediction of 3D geometry using deep neural network architectures.
- **Refinement and Optimization**: Iterative refinement of the reconstructed geometry through loss functions and optimization strategies to reduce reconstruction error.
- **Evaluation Strategy**: Quantitative benchmarking of reconstruction accuracy using standardized geometric and perceptual metrics.

### 2.2. Datasets for 3D Face Reconstruction

Datasets play a critical role in training and evaluating deep learning-based 3D face reconstruction models. Existing datasets [2,27] can generally be categorized into four groups: *3D scan-based datasets*, *image-based datasets with landmark annotations*, *synthetic or 3D morphable model (3DMM)-based datasets*, and *dynamic or multi-view capture datasets*. These datasets provide dense geometric supervision for training and evaluation [27]. Image-based datasets contain large collections of 2D facial images labeled with landmarks or other annotations and are widely used for large-scale training and benchmarking under unconstrained conditions [28,29]. Parametric datasets based on 3D morphable models provide synthetic or model-generated facial geometries with controllable shape, expression, and pose parameters, enabling supervised training and controlled data generation for reconstruction frameworks. Table 3 summarizes the datasets frequently used in the literature, highlighting their modality, scale, annotation type, and availability.

Depth-based datasets provide high-fidelity geometric ground truth and are primarily used for statistical model construction and geometric evaluation [27]. Multi-view or dynamic capture datasets enable motion-consistent modeling and improved occlusion handling, supporting temporal reconstruction and view-consistent synthesis [34]. In contrast, landmark-based datasets provide extensive pose and appearance diversity but only sparse geometric supervision, encouraging learning-based regression methods that rely on statistical priors [29]. Differences in capture modality, annotation density, and dataset scale introduce inherent evaluation biases across benchmarks. High-precision depth-based datasets emphasize geometric accuracy, whereas landmark-based datasets primarily evaluate robustness and generalization under challenging pose, illumination, and expression variation conditions. While Table 3 lists representative datasets used in the literature, these datasets can be further grouped according to their acquisition modality and supervision characteristics.

Table 4 shown below highlights the difference between dataset categories in terms of supervision quality and scalability. Scan-based datasets [15,31] provide highly accurate geometric supervision and are therefore widely used for constructing statistical face models and benchmarking reconstruction accuracy. However, acquiring high-quality 3D scans requires specialized capture systems, controlled lighting environments, and extensive manual processing, which limits the scalability of such datasets in terms of subject diversity and dataset size. In contrast, large-scale in-the-wild image datasets [29,37] are significantly more diverse and easier to collect but typically lack explicit 3D ground truth. As a result, many recent approaches rely on weakly supervised learning strategies that leverage 2D landmarks, photometric consistency, or statistical priors to infer 3D geometry [9]. Synthetic datasets [2] provide an additional alternative by generating large-scale annotated training data through rendering pipelines, although domain gaps between synthetic and real imagery may affect generalization performance [3,7,40]. Dynamic or 4D datasets [41,42] further extend reconstruction research by capturing temporally consistent facial motion, enabling evaluation of expression dynamics and temporal reconstruction; however, they require complex multi-camera acquisition setups.

Due to the practical limitations of large-scale 3D scanning, recent research has increasingly explored hybrid supervision and self-supervised training paradigms that combine limited depth-based datasets with large-scale in-the-wild image collections [7,44]. Such approaches aim to reduce dependence on expensive 3D acquisition pipelines while maintaining reliable geometric reconstruction accuracy, suggesting a promising direction for scalable and practical 3D face reconstruction systems. Many commonly used benchmark datasets, including AFLW2000-3D [2], MICC Florence [30], and NoW [6], primarily focus on variations in pose, illumination, and facial expression but provide limited analysis of performance across demographic attributes such as age, ethnicity, and gender. As a result, reconstruction methods trained on these datasets may exhibit uneven performance across demographic groups when deployed in real-world settings, particularly for individuals from underrepresented populations [17,18]. Densely supervised approaches based on high-quality 3D scan datasets provide strong geometric constraints but are limited in scalability due to high acquisition cost and controlled capture requirements. In contrast, weakly supervised approaches using large-scale in-the-wild images offer greater scalability but often lack precise geometric supervision, leading to reduced structural accuracy [45]. Recent studies in face analysis have highlighted the importance of balanced dataset composition and subgroup evaluation to mitigate potential bias and ensure reliable performance across diverse user populations [46].

#### Hardware Selection on Reconstruction

Beyond descriptive hardware specifications, it is essential to analyze how sensor characteristics directly influence reconstruction accuracy. The choice of acquisition hardware determines the geometric fidelity of captured data, which in turn affects landmark stability, triangulation precision, mesh consistency, and evaluation outcomes [22,47]. These hardware dependent characteristics explain variations in reconstruction performance across benchmarks and highlight the necessity of sensor-aware evaluation that is discussed in detail below.

As summarized in Table 5, different sensing modalities introduce distinction between geometric accuracy, robustness to environmental conditions, and deployment complexity, which directly influence the reconstruction reliability of downstream learning models. In practice, dataset construction often integrates multiple hardware components, including depth sensors, synchronized RGB cameras, structured-light scanners, and dedicated timing units. RGB cameras provide high-resolution texture; however, the absence of direct depth measurements introduces geometric ambiguity during 3D inference [8]. Consequently, reconstruction methods relying solely on RGB imagery often depend heavily on statistical priors or learned shape models to recover plausible geometry [48].

Depth sensors such as active depth sensors provide explicit geometric measurements that reduce depth ambiguity inherent in monocular RGB inputs [53,54]. Structured-light systems offer high spatial precision under controlled lighting, enabling improved mesh alignment and reduced geometric error conditions [55]. However, their sensitivity to ambient illumination and reflective surfaces can introduce missing regions and surface noise, which propagate as reconstruction artifacts [56]. Time-of-flight (ToF) sensors enable real-time capture and improved robustness to lighting variation [50], making them suitable for dynamic facial performance capture. Nevertheless, multipath interference and depth quantization errors reduce fine-scale geometric detail, limiting their effectiveness for high-fidelity expression modeling. Passive multi-view RGB systems reconstruct geometry through triangulation across synchronized camera views.

**Figure 2:**
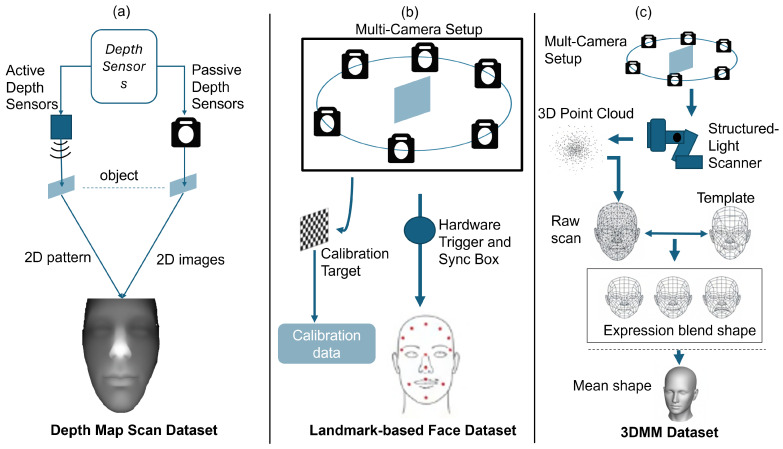
Conceptualization of Calibration and Synchronization as Upstream Constraints in Reconstruction Approaches. (**a**) Approaches relying on active depth sensing technologies, where calibration and synchronization act as upstream constraints influencing the acquisition of depth maps or partial geometry. (**b**) Multi-view landmark-based pipelines that utilize synchronized RGB cameras and precise calibration to capture high-quality 2D facial landmarks and camera parameters. (**c**) High-fidelity 3D morphable model datasets, typically constructed using structured-light or multi-camera scanning systems, followed by template fitting and blendshape generation to obtain identity and expression bases. Red dots: extracted facial landmarks.

Figure 2 shows different acquisition paradigms, including active depth sensing, multi-view RGB capture, and structured-light scanner. This categorization reflects the typical data sources employed in different stages of the 3D face reconstruction pipeline. As discussed earlier, these effects are largely governed by acquisition hardware characteristics.

Consistent multi-angle observations result in lower geometric error and more stable mesh reconstruction [15]. As illustrated in Figure 2c, high-resolution structured-light scanners provide dense point clouds with sub-millimeter precision, enabling accurate ground-truth model construction and statistical shape modeling. Such systems support low scan-to-mesh evaluation but are impractical for real-time deployment due to cost and operational complexity. Mobile sensing platforms, such as Light Detection and Ranging (LiDAR) equipped smartphones, offer portability and rapid acquisition; however, they are often accompanied by sparse and noisy depth measurements [57]. These limitations reduce reconstruction reliability for fine-scale facial features, necessitating stronger reliance on learned priors and refinement networks [52]. Consequently, sensor selection introduces a fundamental distinction between geometric precision, robustness, scalability, and deployment feasibility. Systems relying solely on RGB imagery in Figure 2a often prioritize perceptual realism but exhibit higher geometric error [8], whereas depth-assisted systems achieve improved metric accuracy at the cost of increased hardware complexity and sensitivity to environmental conditions [3]. Active sensing technologies such as structured-light and time-of-flight sensors address this limitation by providing explicit depth measurements. Structured-light systems offer high spatial precision under controlled illumination conditions, enabling accurate surface reconstruction and dense mesh alignment [49,50]. Their performance may degrade in the presence of reflective materials or strong ambient lighting. Time-of-flight sensors enable real-time depth capture and are more robust to illumination variations, making them suitable for dynamic facial capture scenarios, although multipath interference and depth quantization can reduce fine geometric detail [58]. Passive multi-view RGB systems reconstruct geometry through triangulation across synchronized camera views. These systems significantly improve occlusion handling and provide consistent multi-angle observations, which enhances mesh stability and reduces reconstruction error when calibration is accurate. However, their performance strongly depends on precise camera calibration and temporal synchronization [51]. LiDAR sensors provide accurate global depth measurements and are increasingly integrated into mobile devices for rapid 3D capture. While they improve overall geometric consistency, their relatively sparse spatial sampling limits the recovery of fine facial surface details, often requiring additional refinement through learned reconstruction models [52]. Table 6, Table 7, Table 8, Table 9, Table 10 and Table 11 summarize representative acquisition hardware discussed previously and calibration components commonly used in controlled facial capture environments.

**Table 6:** Active depth sensors used in 3D face dataset acquisition. Reported accuracy values correspond to approximate RMS depth error at a distance of ∼1 m under controlled indoor conditions.

Sensor	Technology	Depth Resolution	Accuracy (at ∼1 m)	Remarks
Kinect v1 [59]	Structured Light	640×480	∼8–15 mm	Early RGB-D datasets; sensitive to ambient lighting and limited facial detail
Kinect v2 [60]	Time-of-Flight	512×424	∼2–5 mm	Improved depth stability; commonly used in controlled lab environments
Azure Kinect [61]	Time-of-Flight	Up to 1024×1024	∼1–3 mm	Higher resolution depth; suitable for detailed facial geometry capture
Intel RealSense D415 [62]	Active Stereo (IR)	Up to 1280×720	∼2–6 mm	Compact and widely used; performance degrades with strong IR interference
iPhone LiDAR [57]	Time-of-Flight	∼640×480	∼10–30 mm	Mobile capture; convenient but insufficient for high-fidelity geometry; depth accuracy degrades significantly for small-scale facial features.

Active depth sensors directly measure scene geometry using structured illumination or time-of-flight (ToF) principles. These devices are widely used in early RGB-D datasets and laboratory-based facial capture systems due to their ease of deployment and real-time depth availability. However, depth accuracy varies significantly with capture distance, surface reflectance, and ambient lighting conditions, which we have discussed in Table 6. For instance, Kinect v1 [59] exhibits depth errors of approximately 8–15 mm at 1 m distance, which can introduce significant noise in facial geometry estimation. In contrast, modern time-of-flight sensors such as Azure Kinect provide 1–3 mm accuracy, enabling higher-fidelity ground-truth datasets. Consequently, models trained on datasets acquired with high-precision sensors [33] tend to demonstrate lower reconstruction error compared to those trained on lower-resolution RGB-D datasets [63].

In contrast to active sensors, passive depth estimation relies exclusively on RGB imagery and geometric inference. Stereo and multi-view systems reconstruct depth through triangulation, while photometric stereo and learning-based monocular approaches estimate surface structure from shading or learned priors. Table 7 summarizes systems that offer improved flexibility and scalability.

**Table 7:** Passive depth sensing systems used in 3D face reconstruction datasets.

System	Camera Setup	Depth Principle	Remarks
Binocular Stereo Rig [51,64]	2× RGB	Triangulation	Accuracy depends on texture, calibration, and baseline
Multi-view Stereo (MVS) Rig [65,66]	3–8× RGB	Multi-View Triangulation	More robust to occlusions; increased capture and processing cost
Photometric Stereo Setup [67,68]	1× RGB + Lighting	Shading-Based Normal Estimation	Captures fine surface detail; requires static subjects and controlled lighting
Structure-from-Motion (SfM) Pipeline [69,70]	Moving/Sparse RGB	Feature-Based Reconstruction	Used in uncontrolled settings; sensitive to motion blur and feature quality
Monocular Depth Estimation [71,72]	1× RGB	Learned Depth Prior	Fast but non-metric; unsuitable for precise facial geometry

For dynamic facial capture, spatial calibration alone is insufficient; temporal synchronization is equally critical. Dedicated triggering devices distribute hardware-level signals to ensure simultaneous exposure across all cameras. Synchronization precision directly influences reconstruction accuracy, particularly for expression capture and high-frame-rate sequences, where even minor timing offsets introduce motion-induced geometric artifacts. High-fidelity facial datasets used for statistical modeling and parametric reconstruction typically rely on dedicated scanning systems. These devices provide dense surface measurements with sub-millimeter accuracy under controlled lighting conditions. Such scanners are commonly employed to generate ground-truth identity and expression bases for benchmark datasets as illustrated in Table 8.

**Table 8:** Structured-light scanners used for high-fidelity 3D face dataset construction.

Scanner	Point Density	Accuracy	Remarks
Artec Eva [73]	∼1M points	up to 0.1 mm	Portable structured-light scanner
3dMD Face System [74]	>1M points	∼0.2 mm	Multi-camera photogrammetry system
Creaform [75]	High	up to 0.05 mm	High-resolution structured-light scanner
EinScan Pro [76]	Medium	up to 0.1 mm	Cost-effective structured-light scanner

Representative hardware-based synchronization solutions used in dataset acquisition are summarized in Table 9 which have discussed in detail in Figure 2.

Accurate 3D reconstruction further depends on precise geometric calibration, as summarized in Table 10. Intrinsic and extrinsic parameters define the mapping between 3D world coordinates and image observations, while distortion coefficients compensate for lens-induced nonlinearities. Additional parameters such as baseline distance ensure stable stereo triangulation. For dynamic multi-view capture, temporal calibration becomes equally important as synchronization timestamps must align image streams across cameras to prevent motion-induced inconsistencies.

**Table 9:** Hardware trigger devices used for synchronized multi-camera capture. Reported precision and latency values are approximate and depend on implementation and wiring configuration.

Device	Trigger Precision	Typical Latency	Remarks
Arduino Uno [77]	Millisecond-level	∼1–5 ms	Low-cost triggering; sufficient for static facial capture
ESP32 [78]	Microsecond-level (hardware timer)	∼0.1–1 ms (wired)	Wireless triggering; flexible but requires careful timing control
OptiTrack Trigger Hub [79]	Microsecond-level	<0.1 ms	Professional motion capture setups; high cost but very stable

**Table 10:** Calibration data commonly used in 3D face dataset construction.

Calibration Parameter	Dimensions	Role in 3D Reconstruction
Camera Intrinsics [47,51]	3×3	Defines focal length and principal point for accurate image projection
Camera Extrinsics [51]	3×4	Encodes camera pose for multi-view triangulation and spatial alignment
Lens Distortion Coefficients [47,80]	5–8	Corrects radial and tangential lens distortions
Baseline Distance [64]	Scalar	Determines stereo depth resolution and triangulation stability
Projection Matrix [51]	3×4	Combined intrinsic–extrinsic mapping from 3D world coordinates to image space
Synchronization Timestamps [81]	Scalar	Ensures temporal alignment across synchronized camera streams

To achieve this temporal alignment in practical capture systems, dedicated synchronization units provide centralized clock distribution and deterministic hardware-level exposure control across camera arrays. These devices ensure sub-millisecond timing consistency required for high-frame-rate facial performance capture. Representative synchronization units employed in multi-camera facial dataset acquisition are listed in Table 11. This system-level perspective clarifies why reconstruction performance cannot be evaluated independently of acquisition hardware. The hardware and synchronization components directly influence landmark stability, triangulation accuracy, temporal consistency, and the reliability of geometric supervision in downstream reconstruction pipelines.

**Table 11:** Synchronization units used in multi-camera facial dataset acquisition.

Sync Box	Sync Accuracy	Supported Cameras	Remarks
OptiTrack eSync [79]	Microsecond-level	Multi-RGB/IR	Widely used in motion capture and facial tracking setups
Basler Sync Box [82]	Microsecond-level	Basler industrial cameras	Hardware-level synchronization for high-speed capture
FLIR Sync Module [83]	Microsecond-level	FLIR machine vision cameras	Common in research-grade multi-camera rigs
Custom FPGA Sync Box [84]	Sub-microsecond	Custom	High-precision hardware timing; complex implementation

### 2.3. Preprocessing

Preprocessing constitutes an essential preliminary stage in 3D face reconstruction pipelines, ensuring that subsequent modules operate on a normalized and well-defined facial region. Given that many datasets and real-world inputs contain background clutter, multiple subjects, and varying image resolutions, preprocessing is required to isolate the target face and reduce variability unrelated to facial geometry. Typical steps include face detection, cropping, and geometric normalization [85]. Face detection algorithms are employed to localize the facial region within the input image [86], after which the detected face is cropped and resized to a canonical resolution suitable for neural network input. This normalization step improves robustness to scale variations and facilitates stable training across diverse datasets [23]. In many pipelines, image alignment is further applied using detected facial landmarks to enforce consistent orientation, reducing the impact of in-plane rotation and translation [87]. Additionally, some approaches incorporate background removal or facial segmentation during preprocessing [88], particularly in unconstrained or “in-the-wild” scenarios. By suppressing non-facial pixels, segmentation-based preprocessing can reduce noise and improve landmark localization and reconstruction accuracy. While not universally required, such techniques are increasingly adopted in recent works [3,89] to enhance robustness under challenging lighting conditions, occlusions, or complex backgrounds. Although preprocessing is often treated as an auxiliary step, its quality has a direct influence on downstream tasks such as landmark detection, pose estimation, and 3D model fitting [8]. Consequently, careful preprocessing is a critical enabler for accurate and stable 3D face reconstruction and forms the foundation upon which the remaining stages of the pipeline are built.

### 2.4. Facial Landmark Detection

**Figure 3:**
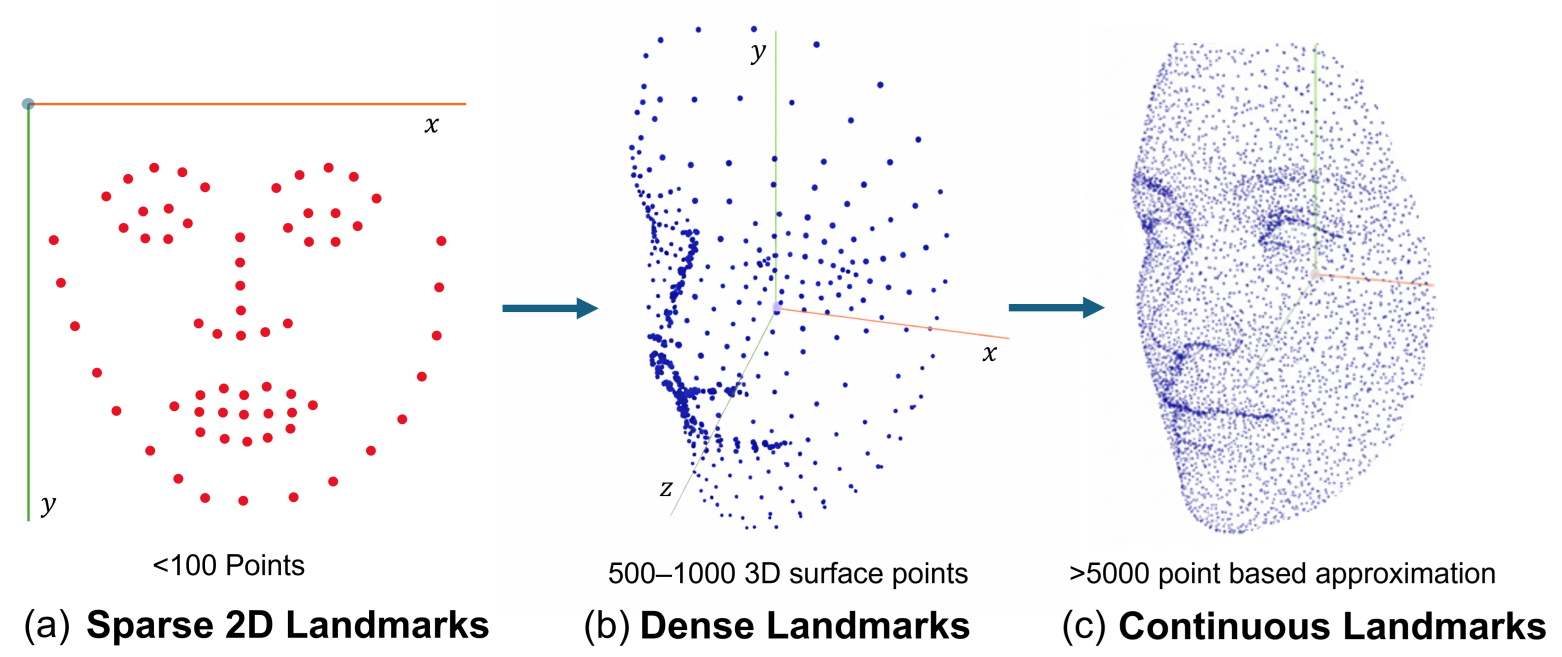
Progressive Geometric Representations Commonly Used in 3D Face Reconstruction Pipelines: Sparse 2D facial landmarks, (**a**) provide coarse geometric constraints, which are lifted into sparse 3D surface samples, (**b**) typically consisting of 500–1000 points. These samples are subsequently refined into dense surface approximations, (**c**) enabling detailed facial geometry reconstruction through mesh- or graph-based refinement. Sparse landmark-based representations have historically been used to provide coarse geometric constraints [2,90], while recent approaches progressively refine sparse estimates into dense surface reconstructions using mesh-based or graph-based models [7,25].

Facial landmark detection is essential for providing structured geometric correspondences between 2D image observations and 3D facial representations [87,90]. Landmarks represent semantically meaningful facial keypoints such as eye corners, nose tips, mouth contours, and jawlines that remain relatively consistent across different identities and expressions. By anchoring the reconstruction process to these stable reference points, landmark detection significantly reduces the ambiguity inherent in monocular 2D-to-3D inference [2,91]. In most deep learning-based reconstruction pipelines, detected landmarks serve as geometric constraints that guide the estimation of 3D face shape and pose. These constraints are particularly important when fitting parametric reconstruction models [4,16], where landmark correspondences are used to align the 3D model with the input image and to regress shape, expression, and pose parameters. Accurate landmark localization improves convergence during optimization and directly impacts the fidelity of the reconstructed geometry [14]. In Figure 3a sparse landmark configurations typically refer to the number of 2D keypoints used for supervision, whereas dense representations in Figure 3b refer to the number of 3D vertices or surface samples used to model facial geometry.

As described in Table 12 landmark configurations vary across existing approaches and are typically categorized as *sparse* or *dense* [87,90]. Sparse landmark sets, commonly consisting of 5 to 68 keypoints following standard protocols such as AFLW or IBUG, are computationally efficient and sufficient for estimating coarse facial structure and head pose [2,90]. However, they often lack the spatial resolution required to capture fine-grained surface details, such as cheek contours or subtle expression variations [9]. Dense or continuous correspondence representations, in contrast, provide hundreds to thousands of facial surface points through UV-space regression or mesh-based supervision [7,91]. These configurations enable more precise modeling of local geometric details and are particularly beneficial for high-fidelity reconstruction and expression-aware modeling, albeit at increased computational cost [3,92]. Recent works explore hybrid strategies that combine sparse landmarks for global alignment with dense correspondences or learned refinement modules for local detail recovery [7]. In summary, landmark selection remains a key design decision that influences both the performance and scalability of 3D face reconstruction systems.

### 2.5. Pose and Camera Modeling

**Figure 4:**
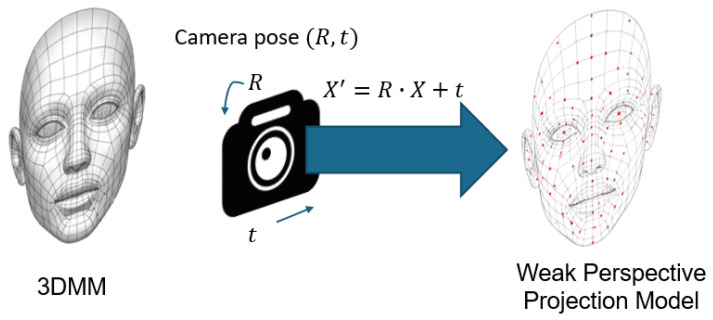
3D Morphable Model (3DMM) Formulation and Weak-Perspective Projection. A parametric 3D face shape is represented as a linear combination of identity and expression bases. The reconstructed geometry is transformed using a rigid pose defined by rotation and translation, followed by weak-perspective projection onto the image plane. This formulation enables efficient camera modeling and landmark reprojection for self-supervised optimization in monocular 3D face reconstruction pipelines.

Pose and camera modeling form an essential component of 3D face reconstruction pipelines as they establish the spatial relationship between the reconstructed 3D facial geometry and the 2D input image [4,51]. Figure 4 illustrates the 3DMM formulation and weak-perspective projection model commonly used in parametric reconstruction frameworks. Accurate estimation of head pose and camera parameters is essential for aligning the 3D face representation with image observations and for enabling meaningful reprojection-based supervision during training [3,92]. Such modeling ensures geometric consistency between the predicted 3D structure and 2D landmarks or pixel-level correspondences, thereby stabilizing optimization and improving reconstruction fidelity.

In most reconstruction frameworks, facial geometry is represented using a parametric reconstruction model such as a 3D morphable model (3DMM) [4,16]. The 3D facial shape is typically expressed as a linear combination of identity and expression bases:

(1) $$S\left(\alpha ,\beta \right)=\bar{S}+B_{\mathrm{id}}\alpha +B_{\exp}\beta ,$$

where $\bar{S}$ denotes the mean face, $B_{\mathrm{id}}$ and $B_{\exp}$ represent the identity and expression bases, and α and β are the corresponding coefficient vectors. The resulting shape $S\in R^{3N}$ consists of *N* stacked 3D vertices. This formulation originates from the statistical modeling of facial shape variation introduced in [4] and later extended to expression modeling in [15,16].

To place the reconstructed face in a common coordinate system, a rigid transformation is applied to the 3D geometry [51]:

(2) $$X^{\prime }=RX+t,$$

where R∈SO(3) is the rotation matrix encoding head pose (yaw, pitch, and roll), $t\in R^{3}$ is the translation vector, and $X\in R^{3}$ denotes a 3D point on the facial surface. Rigid pose estimation plays a central role in 3DMM fitting and alignment [2,92] as errors in pose may be absorbed into incorrect shape deformation during optimization.

Camera modeling defines how transformed 3D points are projected onto the 2D image plane. Most deep learning-based 3D face reconstruction methods adopt a weak-perspective (scaled orthographic) projection model due to its simplicity and numerical stability [3,92]:

(3) $$x=s\;\Pi \;\left(RX+t\right)+t_{2D},$$

where $\Pi \left({\left[X,Y,Z\right]}^{⊤}\right)={\left[X,Y\right]}^{⊤}$ denotes orthographic projection, *s* is a global scale factor, and $t_{2D}\in R^{2}$ represents 2D translation. This model is widely used in parametric reconstruction-based pipelines and provides a good approximation for near-frontal or moderately posed faces.

For scenarios involving large pose variations, perspective projection models derived from classical camera geometry are employed [51]:

(4) $$\tilde{x}=K\left(RX+t\right),\;x={\left[\frac{\tilde{x}}{\tilde{z}},\;\frac{\tilde{y}}{\tilde{z}}\right]}^{⊤},$$

where K denotes the camera intrinsic matrix and $\left(\tilde{x},\tilde{y},\tilde{z}\right)$ are homogeneous projected coordinates. Perspective models introduce additional parameters but allow more accurate modeling with significant viewpoint changes.

Pose and camera parameters are commonly optimized through landmark reprojection losses that measure the discrepancy between detected 2D landmarks and projected 3D landmarks [3,91]. Let ${\left\{l_{i}\right\}}_{i=1}^{M}$ denote detected 2D landmarks and ${\left\{L_{i}\right\}}_{i=1}^{M}$ the corresponding 3D landmarks extracted from the reconstructed mesh S. A typical reprojection loss is defined as

(5) $$L_{\mathrm{lm}}=\frac{1}{M}\sum_{i=1}^{M}\|l_{i}-\Pi \left(RL_{i}+t\right){\|}_{2}^{2},$$

where Π(·) denotes either the weak-perspective projection in Equation (3) or the full perspective projection in Equation (4). The 3D landmark set $\left\{L_{i}\right\}$ is typically defined by selecting predefined semantic vertex indices within the parametric facial mesh. This loss directly couples pose, camera parameters, and facial geometry, enabling coherent optimization within end-to-end reconstruction pipelines.

Overall, pose and camera modeling provide the geometric foundation that links 3D face representations with 2D image observations. Accurate modeling at this stage is essential for stable optimization, reliable reprojection-based supervision, and high-fidelity 3D face reconstruction. In parametric reconstruction, a potential challenge arises from the coupling between pose parameters and shape coefficients during optimization. Errors in the estimated rotation matrix may be partially compensated by adjustments in the shape parameters, resulting in pose-related artifacts in the reconstructed geometry [4,9]. This pose-shape ambiguity occurs because multiple parameter combinations can produce similar 2D projections for the reprojection loss formulation.

To mitigate this effect, many reconstruction frameworks incorporate additional constraints beyond the landmark reprojection objective in Equation (2). Regularization terms on the shape and expression coefficients restrict the solution to the learned statistical distribution of the 3D morphable model [2,4]. Furthermore, multi-view supervision, dense photometric losses, or temporal consistency constraints can provide additional geometric signals that reduce parameter entanglement [7]. Although reprojection losses help stabilize the joint estimation of pose and geometry, complete disentanglement remains challenging, and improving parameter decoupling remains an active research direction in parametric face reconstruction [3,9].

### 2.6. Primary Reconstruction

Primary reconstruction constitutes the core computational stage in which a 2D facial image captured by an RGB sensor is mapped to a 3D facial representation. As illustrated in Figure 3, reconstruction strategies often transition from sparse landmark-based representations to dense mesh refinement mechanisms. In modern deep learning-based pipelines, this process is predominantly realized through encoder–decoder architectures that learn a nonlinear mapping from image space to geometric parameters or surface representations [3,91,92]. These reconstruction strategies align with the taxonomy defined in Section 1.4.

#### 2.6.1. Parameter Regression via Encoder Networks

In parametric reconstruction frameworks, the primary objective of the encoder network is to extract a high-level latent representation from the input image and regress the coefficients of a facial model, such as a 3D morphable model (3DMM) [4,16]. Convolutional Neural Networks (CNNs) are commonly employed as encoders due to their strong feature extraction capabilities. Popular backbone architectures include ResNet-50 [98], VGG-16 [99], and MobileNet-V2 [13]. Earlier reconstruction approaches relied on iterative optimization procedures to estimate model coefficients, often minimizing landmark reprojection and photometric errors [4,100]. Although accurate, such methods were computationally expensive and unsuitable for real-time applications. In contrast, contemporary deep learning methods directly regress 3DMM parameters in a single forward pass [3,92], significantly improving inference speed and scalability. The encoder typically outputs a latent vector comprising identity coefficients (α), expression coefficients (β), and, in some cases, texture or appearance parameters (δ), which are subsequently used to synthesize the 3D face geometry [91,92].

Although 3D morphable models provide a compact representation of facial geometry, their shape and expression spaces are typically defined by linear statistical bases learned from limited training scans [4,15]. As a result, extremely exaggerated or rare facial expressions may lie outside the span of the learned distribution. When regression networks predict expression coefficients outside the training distribution, unrealistic geometric deformations may occur. To mitigate this issue, most reconstruction frameworks incorporate regularization terms that constrain the predicted expression coefficients within the plausible range of the statistical model [4]. In addition, recent learning-based approaches introduce nonlinear deformation models, expression latent spaces, or corrective displacement networks that extend the expressive capacity of traditional parametric reconstruction representations. These strategies allow moderate extrapolation beyond the original training distribution while maintaining geometrically plausible facial structures. In regression-based frameworks, these regularization mechanisms are typically applied during training to constrain predicted expression coefficients within the learned distribution, thereby reducing the risk of implausible extrapolation at inference time [7,9]. However, despite these constraints, accurately modeling highly exaggerated expressions remains challenging and continues to be an open problem in current 3D face reconstruction methods.

#### 2.6.2. Decoders and Differentiable Rendering in Parametric Reconstruction

Recent methods such as DECA [7] further introduce expression-specific latent representations to improve modeling of fine-grained facial deformations. Once the facial parameters are regressed, a decoder or a differentiable rendering module transforms these coefficients into a 3D mesh or a projected 2D representation. In linear parametric reconstruction-based pipelines, the decoder reconstructs the facial surface by combining the regressed coefficients with pre-computed basis matrices, such as those provided by the Basel Face Model [4,27], to obtain final vertex positions. More recent approaches extend beyond linear decoders by employing neural decoders that learn nonlinear deformations of the facial surface [25]. Differentiable rendering plays a key role in these frameworks, enabling the projection of reconstructed 3D geometry back onto the 2D image plane in a fully differentiable manner [101,102]. This allows the computation of pixel-wise, landmark-based, or perceptual loss functions. In practice, differentiable rendering can be implemented through several computational strategies depending on the underlying scene representation. Mesh-based reconstruction methods [3,91] commonly employ differentiable rasterization, where projected vertex positions, visibility, and shading are computed on the image plane while maintaining gradients with respect to camera pose, lighting parameters, and mesh geometry [101]. This enables pixel-level photometric and landmark reprojection losses to directly update the 3D parameters during optimization.

Alternatively, volumetric rendering approaches represent the scene as a continuous density and radiance field. In these methods discussed in Section 2.6.5 below, color contributions are integrated along camera rays using differentiable volume rendering equations, allowing gradients to propagate through sampled spatial locations and enabling joint optimization of geometry and appearance parameters. More recently, *point-based rendering techniques* such as Gaussian splatting [103] have been employed to approximate geometry using collections of anisotropic primitives. These primitives are projected to the image plane and blended through differentiable screen-space operations, enabling efficient optimization while maintaining gradient flow with respect to primitive position, shape, and radiance parameters.

#### 2.6.3. Direct Geometry Estimation

Beyond parametric reconstruction, several modern frameworks explore direct geometry estimation strategies that bypass explicit parametric reconstruction [40]. In these approaches, neural networks predict geometric representations directly from the input image. For example, position map regression methods estimate a dense 2D map in which each pixel encodes the corresponding 3D coordinates of the facial surface [8], enabling high-resolution reconstruction without reliance on a linear basis. Graph-based approaches further model the facial surface as a mesh and apply Graph Convolutional Networks (GCNs) [104] to operate directly on mesh vertices [25,96]. Such methods, including DF2Net [5], capture fine-grained geometric details by exploiting the intrinsic connectivity of the facial mesh [5]. While direct geometry estimation can provide increased expressiveness, it often requires stronger supervision and greater computational resources [3,92] compared to parametric reconstruction-based pipelines.

#### 2.6.4. GAN-Based 3D Face Reconstruction

Generative adversarial networks (GANs) have been widely adopted to improve the visual realism of reconstructed faces [105]. Several reconstruction approaches leverage pretrained generative models to estimate facial appearance and texture, enabling pipelines to synthesize highly photorealistic facial images [106,107,108]. In many cases, GAN priors are integrated with 3D morphable model (3DMM) parameter estimation. For example, GANFit [11] employs a pretrained GAN generator as a prior for facial texture while optimizing 3DMM parameters to match the input image. Through adversarial and perceptual losses, these methods capture high-frequency texture details such as skin appearance, illumination effects, and identity-related features [109]. However, despite producing visually convincing renderings, GAN-based reconstruction methods do not necessarily guarantee accurate geometric recovery. This limitation arises because adversarial and perceptual losses are typically computed in image space, encouraging realism in the rendered image rather than explicitly constraining the underlying 3D shape [3]. Consequently, multiple geometric configurations may produce visually similar renderings, introducing ambiguity in the recovered facial structure [9]. In contrast, methods that incorporate explicit geometric supervision such as vertex-level losses, depth constraints, or scan-to-mesh alignment tend to achieve more reliable structural accuracy [8]. This distinction explains why GAN-based approaches can achieve strong perceptual realism while exhibiting higher geometric reconstruction errors.

#### 2.6.5. Neural Rendering and Radiance-Field-Based Approaches

Recent advances in neural rendering have introduced radiance-field-based representations that enable photorealistic modeling of human faces and novel view synthesis [110]. Unlike conventional reconstruction pipelines that explicitly recover geometric structure through parametric models or dense mesh regression, neural rendering approaches learn implicit volumetric representations that jointly encode geometry, appearance, and view-dependent effects.

**Figure 5:**
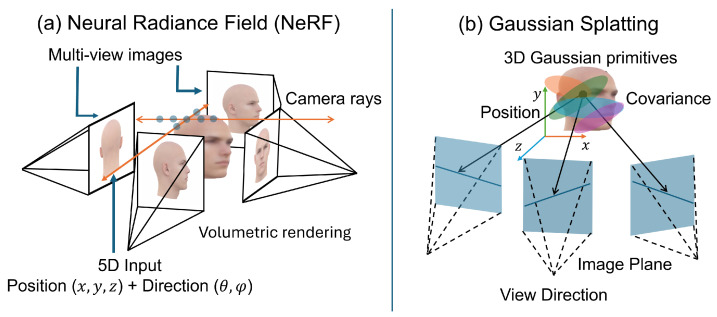
Conceptual Comparison between Neural Rendering Paradigms. (**a**) Neural radiance fields (NeRFs) reconstruct scenes as continuous volumetric functions learned from multiple camera views, where camera rays sample density and color along the viewing direction. (**b**) Gaussian splatting represents scenes using anisotropic 3D Gaussian primitives defined by position, covariance (shape), and radiance, which are projected onto the image plane using screen-space splatting for efficient rendering.

As shown in Figure 5a, neural radiance fields (NeRFs) represent scenes as continuous volumetric functions parameterized by neural networks that map spatial coordinates and view directions to density and radiance values [110]. When applied to facial modeling, NeRF-based approaches synthesize high-quality novel views while capturing fine-scale texture details and complex illumination effects. Unlike explicit mesh-based differentiable rendering pipelines, volumetric neural rendering methods represent scenes using continuous density and radiance fields and integrate color contributions along camera rays through volumetric rendering [111].

Gaussian splatting [103] approximates scenes using sets of anisotropic Gaussian primitives whose positions, covariance parameters, and radiance properties are optimized from multi-view observations, as illustrated in Figure 5b. Compared to volumetric radiance fields, Gaussian splatting enables real-time rendering and improved scalability, making it attractive for dynamic human modeling. Nevertheless, similar to NeRF-based methods, Gaussian splatting primarily emphasizes view synthesis quality rather than precise metric reconstruction of facial geometry. Neural implicit surface representations further bridge geometric modeling and neural rendering by encoding signed distance functions or occupancy fields within neural networks [111,112]. While Gaussian splatting-based approaches produce smoother surface reconstructions than volumetric radiance fields, they typically rely on multi-view supervision and computationally intensive optimization procedures [113].

Overall, neural rendering paradigms prioritize photorealistic appearance and view-consistent rendering [110,114], whereas classical parametric and dense regression methods focus more directly on geometric accuracy and efficient inference [4,7]. Consequently, neural rendering techniques are particularly well suited for applications such as avatar synthesis, telepresence, and virtual reality.

#### 2.6.6. Dynamic Facial Reconstruction

While traditional 3D face reconstruction focuses on recovering geometry from static images, recent research has shifted toward dynamic 4D facial modeling from video sequences. This transition requires models to capture rapid expressions and head motion while maintaining temporal consistency [115]. Unlike static pipelines, dynamic frameworks enforce coherence across frames using temporal modeling strategies such as recurrent networks, temporal convolutions, or sequence-based optimization to stabilize predictions and reduce frame-to-frame jitter [10,116]. In many cases, these methods extend earlier parametric reconstruction pipelines, augmenting them with temporal constraints and additional loss terms to ensure smooth transitions in pose and shape [7]. Despite its importance for applications such as telepresence and virtual reality [117], dynamic reconstruction remains challenging due to occlusions, illumination variations, and rapid facial motion. To address these issues, many recent approaches disentangle identity-related shape parameters from time-varying expression coefficients, enabling a stable identity representation while tracking facial dynamics over time [118].

#### 2.6.7. Visual Taxonomy

**Figure 6:**
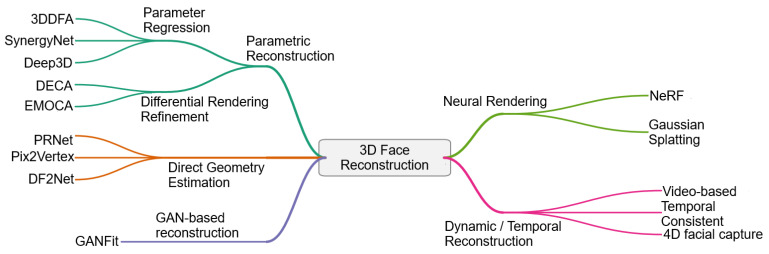
Taxonomy of Deep Learning-Based 3D Face Reconstruction Methods. Existing approaches can be broadly categorized into parametric reconstruction [2,3,12], direct geometry estimation [7,18], GAN-based reconstruction [11], neural rendering [110,114], and dynamic/temporal reconstruction [115].

An overview of the major methodological paradigms in deep learning-based 3D face reconstruction is illustrated in Figure 6.

Parametric reconstruction methods estimate coefficients of statistical face models through encoder-based parameter regression, often followed by refinement via differentiable rendering. Direct geometry estimation approaches instead predict dense facial geometry directly from images using position-map regression or mesh-based learning frameworks. GAN-based reconstruction methods incorporate adversarial priors to enhance photorealistic appearance while typically leveraging statistical or parametric facial representations. Neural rendering frameworks, including neural radiance fields (NeRFs) and Gaussian splatting [103], model facial appearance and geometry using implicit volumetric or point-based representations learned from multi-view observations. Finally, dynamic and temporal reconstruction methods extend these paradigms to video sequences, enabling temporally consistent estimation of facial geometry and expressions across frames.

### 2.7. Refinement and Optimization

Refinement and optimization represent the final stage of the 3D face reconstruction pipeline, where the initial geometry and parameters estimated during primary reconstruction are further improved to enhance geometric accuracy and visual fidelity [3,7,91]. This stage is essential for bridging the gap between coarse facial structure obtained through parametric regression and high-quality reconstructions that capture fine-grained surface details and identity-specific characteristics.

#### 2.7.1. Loss Function Configurations

Optimization in modern 3D face reconstruction frameworks is typically guided by multi-task loss functions that jointly supervise different aspects of the reconstruction process. Rather than relying on a single objective, contemporary approaches combine multiple complementary losses to stabilize training and improve convergence. Common loss formulations encountered in modern 3D face reconstruction frameworks include:

- **Landmark Reprojection Loss** ($L_{\mathrm{lm}}$): Minimizes reprojection error between detected 2D landmarks and projected 3D landmarks [2,92]. This loss provides strong structural constraints and stabilizes pose and shape estimation.
- **Photometric Consistency Loss** ($L_{\mathrm{photo}}$): Penalizes pixel-wise differences between the input image and rendered reconstruction [3,91]. Common in weakly supervised or self-supervised pipelines.
- **Perceptual Loss** ($L_{\mathrm{perceptual}}$): Compares deep feature representations extracted from pre-trained networks to preserve identity-specific characteristics [3,119]. Unlike pixel-based losses, perceptual supervision captures high-level semantics.
- **Shape Regularization Loss** ($L_{\mathrm{shape}}$): Constrains identity parameters to remain within the learned 3DMM shape distribution [4,16].
- **Expression Regularization Loss** ($L_{\mathrm{expression}}$): Controls expression parameters (e.g., β) to balance expressive flexibility. Regularizes expression parameters to avoid implausible deformations [7,15].
- **Adversarial Loss**: Introduced in GAN-based frameworks to enhance visual realism [11,120]. While effective for fine-scale detail enhancement, adversarial training increases optimization complexity.
- **Dense or Vertex-based Loss**: Supervises vertex-level distances or dense correspondences between reconstructed and ground-truth meshes [8,25].

In most deep learning-based 3D face reconstruction frameworks, the overall training objective is formulated as a weighted combination of multiple loss terms that supervise different aspects of the reconstruction process. A general form of the optimization objective can be expressed as

(6) $$L=\lambda_{photo}L_{photo}+\lambda_{land}L_{lm}+\lambda_{perc}L_{perceptual}+\lambda_{shape}L_{shape}+\lambda_{expr}L_{expression}$$

where the weighting coefficients $\lambda_{i}$ control the relative importance of each supervision signal and are typically treated as hyperparameters that balance loss constraints. Photometric loss encourages pixel-level consistency between the rendered reconstruction and the input image, while landmark losses enforce geometric alignment of key facial points. Perceptual losses capture high-level semantic similarity using deep feature spaces, and regularization terms constrain the reconstructed parameters within plausible facial shape and expression distributions.

The choice of loss weighting significantly influences the reconstruction behavior. Increasing photometric or perceptual loss weights generally improves visual realism and texture fidelity, whereas stronger landmark or geometric supervision tends to produce more accurate facial geometry. However, excessive reliance on image-based losses may lead to ambiguous 3D solutions due to the inherent depth ambiguity of monocular images.

In practice, multiple complementary losses are jointly optimized to balance geometric accuracy, perceptual realism, and training stability, as illustrated in Table 13. As demonstrated by Wu et al. [12], the combination of multiple loss terms contributes to stable training and improved reconstruction accuracy. Gao et al. [94] further extend this by introducing loss for individual modules, each with independent responsibility. Table 13 highlights clear differences in supervision strategies across reconstruction paradigms. Early parametric regression methods such as 3DDFA [2], SynergyNet [12], and RingNet [6] rely primarily on sparse landmark constraints combined with shape and expression regularization, resulting in relatively simple objective compositions. In contrast, differential rasterization frameworks, including DECA [7], EMOCA [18], Faceverse [121], and HRN [122], incorporate photometric and perceptual losses to enhance identity preservation and fine-detail recovery. Dense geometry approaches such as PRNet [8] and Pix2Vertex [96] emphasize vertex-level supervision for improved surface fidelity, while GANFit [11] employs one of the most diverse objective configurations by integrating adversarial, perceptual, and photometric constraints to jointly optimize geometric accuracy and visual realism.

#### 2.7.2. Iterative Refinement and Feedback Networks

To achieve high reconstruction fidelity, many frameworks employ iterative refinement strategies in which reconstruction outputs are progressively improved through feedback mechanisms [7,91]. In such pipelines, the errors computed by the loss functions are propagated back into the network, allowing for gradual adjustment of vertex positions and model parameters. Beyond iterative updates, dedicated refinement modules may be introduced to operate on intermediate reconstructions. For example, the DF2Net [5] architecture utilizes a framework boosted by a fine-to-finer refinement network as a calibrated module for highly accurate representation. While these refinement techniques significantly improve reconstruction quality, they often increase computational complexity, typically making CUDA compatibility a basic hardware requirement for feasible runtimes.

#### 2.7.3. Texture and Mesh Validation

The final phase of refinement focuses on validating the reconstructed facial model against the original input image to ensure geometric and appearance consistency. When texture modeling is supported, color information from the input image is projected onto the reconstructed mesh through differentiable rendering [101,102]. Additional optimization steps may be applied to minimize residual discrepancies between rendered projections and detected landmarks [3,91]. Through the combined use of multi-term losses, iterative feedback, and texture-aware validation, the refinement stage plays a pivotal role in producing accurate, stable, and visually convincing 3D face reconstructions suitable for downstream applications.

### 2.8. Evaluation Metrics and Comparative Analysis

Evaluation of 3D face reconstruction methods is inherently challenging due to the coexistence of multiple representation paradigms and evaluation objectives. Different methods optimize either geometric accuracy or perceptual realism, making direct comparison non-trivial. Consequently, benchmarking requires a combination of geometric, perceptual, and system-level metrics interpreted with consistent evaluation protocols.

#### Common Metrics

A variety of quantitative metrics are used to evaluate 3D face reconstruction from different perspectives:

- **Normalized Mean Error (NME)**: Measures landmark-based alignment accuracy and is widely used on AFLW2000-3D [2].
- **Root Mean Square Error (RMSE)**: Quantifies point-to-plane or point-to-point distance between reconstructed meshes and ground-truth scans, commonly reported on MICC Florence [30].
- **Scan-to-Mesh Error (Median/Mean/Std)**: Used in the NoW Challenge [6] to measure per-vertex geometric deviation from high-quality 3D scans.
- **PSNR (Peak Signal-to-Noise Ratio)**: Measures pixel-wise fidelity between rendered reconstructions and reference images. It is widely used in image restoration and rendering evaluation [123].
- **SSIM (Structural Similarity Index)**: Assesses perceptual similarity by comparing local luminance, contrast, and structural consistency between images [124].
- **LPIPS (Learned Perceptual Image Patch Similarity)**: Computes perceptual distance using deep feature representations extracted from pre-trained networks, providing improved correlation with human visual judgment [26].
- **Absolute Distance (ABS)**: Quantifies geometric reconstruction accuracy by measuring the average Euclidean distance between reconstructed mesh vertices and ground-truth 3D scans [6,30].

To provide a structured overview of these evaluation criteria, Table 14 categorizes commonly used metrics according to their evaluation objectives and highlights their dependence on sensing modalities and hardware configurations.

Table 14 summarizes the relationship between evaluation metrics, sensing modalities, and hardware dependencies, highlighting that reconstruction quality is assessed from multiple, often complementary perspectives. Geometric metrics remain the primary measure of structural accuracy. They quantify deviations between reconstructed geometry and ground-truth facial scans using metrics such as Normalized Mean Error (NME), Root Mean Square Error (RMSE), and scan-to-mesh distance [2,27]. Perceptual metrics, including PSNR, SSIM, and LPIPS, evaluate the visual realism and texture fidelity of rendered outputs [26,124]. These metrics are particularly relevant for rendering-oriented approaches such as neural radiance fields (NeRFs) [110] and Gaussian splatting [103], where high-quality view synthesis is prioritized. However, strong perceptual performance does not necessarily imply accurate underlying geometry as visually plausible renderings may be achieved despite structural inaccuracies. Landmark-based metrics measure the consistency between projected 3D landmarks and annotated 2D keypoints [28,37]. Efficiency metrics such as runtime, memory consumption, and frames per second (FPS) assess practical deployability and hardware constraints. Importantly, direct comparison across methods remains challenging due to variations in evaluation protocols. Metrics with identical names may be computed with different alignment procedures (e.g., rigid Iterative Closest Point (ICP) alignment for scan-to-mesh registration versus landmark-based alignment), normalization strategies, or evaluation regions [3,125]. These inconsistencies can lead to systematic discrepancies in reported performance and must be considered when interpreting benchmark results. The results summarized in the next section are aggregated from the original publications and therefore reflect their respective evaluation protocols, where geometric errors are typically computed after alignment procedures such as ICP or landmark-based registration, and should be interpreted alongside perceptual metrics for a comprehensive assessment of reconstruction quality.

## 3. Benchmark Analysis and Quantitative Comparison

To provide an evidence-driven benchmark analysis of representative 3D face reconstruction methods, this section consolidates quantitative results reported in prior studies. The analysis aims to identify systematic performance patterns across architectural paradigms, with particular emphasis on geometric accuracy, perceptual fidelity, and computational efficiency. In addition to accuracy-based evaluation, we examine hardware trends to contextualize the practical deployment of reconstruction systems.

**Figure 7:**
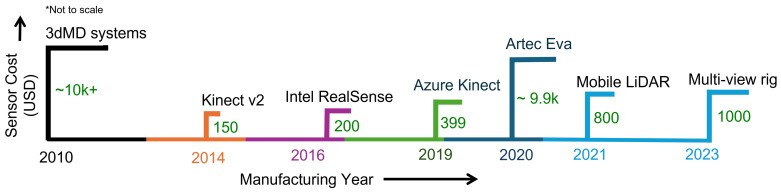
Evolution of Sensing Hardware Cost for 3D Face Reconstruction Systems. Early structured-light scanners required high-cost setups, while consumer depth sensors significantly reduced cost. Color transitions along the timeline denote the evolution of sensing paradigms across successive sensor generations, illustrating key technological shifts in hardware development.

Figure 7 illustrates the evolution of sensing hardware cost for 3D face reconstruction systems. Early structured-light scanners required high-cost acquisition setups, often exceeding 10,000 USD, limiting their use to controlled laboratory environments. The introduction of consumer depth sensors, such as Kinect, significantly reduced hardware cost to a few hundred dollars, enabling wider accessibility and large-scale data collection. More recent developments reveal a divergence in sensing paradigms. Mobile sensing solutions offer low-cost and practical deployment, whereas high-precision professional scanners remain an expensive means of achieving superior geometric accuracy. This trend highlights a fundamental trade-off between cost, accessibility, and reconstruction fidelity, indicating that advances in sensing hardware have improved usability without fully eliminating performance constraints.

Table 15 presents a quantitative comparison of reconstruction accuracy across sensing modalities. Structured-light systems generally report lower geometric error under controlled acquisition conditions due to high-precision depth capture. Multi-view RGB systems often reduce reconstruction error through triangulation and improved occlusion handling, although at the cost of increased system complexity and reduced inference speed. Time-of-flight sensors provide a balance between real-time performance and geometric accuracy, while monocular RGB methods exhibit higher reconstruction error due to inherent depth ambiguity despite their scalability and efficiency. Mobile LiDAR-based sensing enables practical deployment but introduces higher geometric error due to sparse depth sampling and sensor noise. These results suggest that reconstruction performance is strongly influenced by acquisition modality rather than model design alone. The values are aggregated from studies conducted with different datasets, evaluation protocols, and alignment procedures, and the reported ranges are interpreted as indicative trends. In addition to this system-level analysis, Table 16 summarizes reported hardware requirements and runtime characteristics, further clarifying the trade-offs between reconstruction accuracy and practical deployment.

The results reveal clear performance trends across different reconstruction paradigms, highlighting a strong relationship between geometric accuracy and computational efficiency. Classical optimization-based approaches such as 3DMM fitting exhibit the highest reconstruction error (8.0% NME) while also incurring the largest computational cost (2000–5000 ms per image), confirming their limited suitability for real-time applications. In contrast, learning-based methods significantly reduce reconstruction error while improving inference speed. Among early deep regression approaches, 3DDFA [2] achieves moderate performance (5.4% NME at 25.5 FPS), whereas dense correspondence methods such as PRNet [8] improve accuracy (3.6% NME) while achieving high throughput (102 FPS). However, geometric estimation-based regression does not guarantee optimal geometric fidelity, as evidenced by Pix2Vertex [96] (4.0% NME) despite its high efficiency (209 FPS). More recent parametric and hybrid approaches demonstrate a better balance between accuracy and speed. Methods such as Deep3DFaceRecon [3] (2.8% NME, ∼100 FPS) and HRN [122] (2.6% NME, ∼180 FPS) achieve strong geometric accuracy while maintaining real-time performance, indicating the effectiveness of efficient CNN-based regression architectures. Similarly, FaceVerse [121] (2.5% NME, 30 FPS) and EMOCA [18] (3.0% NME, 50 FPS) show that parametric models augmented with expression modeling or differentiable refinement can improve reconstruction quality without substantial runtime penalties. The lowest reconstruction errors are observed in methods that incorporate stronger geometric priors or iterative refinement. RingNet [6] achieves one of the best results (1.8% NME) while still operating in near real-time (65 FPS), demonstrating the advantage of FLAME-based parametric modeling. Optimization-driven methods such as GANFit [11] further improve accuracy (2.0% NME) but at a significantly higher computational cost (2000 ms per image). Similarly, graph-based refinement approaches such as MGCNet [127] (2.5% NME, ∼3 FPS) achieve competitive accuracy but remain computationally expensive.

While Table 16 represents computational characteristics of state-of-the-art methods, practical deployment on edge devices introduces additional constraints beyond runtime and accuracy. Several regression-based methods (e.g., HRN [122], Deep3DFaceRecon [3], and PRNet [8]) demonstrate real-time performance and CPU feasibility, though their deployment may still be constrained. In contrast, optimization-based and refinement-heavy approaches (e.g., GANFit [11] and MGCNet [127]) remain impractical for edge deployment due to their high computational cost and latency. These observations highlight a critical trade-off between reconstruction fidelity and deployability. Lightweight architectures improve scalability and real-time performance but may sacrifice fine geometric detail, whereas high-fidelity methods require substantial computational resources.

### 3.1. Quantitative Benchmarking Analysis

Quantitative evaluation of 3D face reconstruction methods was performed using standardized facial benchmarks and task-specific metrics that measure structural accuracy and photometric consistency. Geometric fidelity is commonly evaluated using Normalized Mean Error (NME), Root Mean Square Error (RMSE), and scan-to-mesh error reported on datasets such as AFLW2000-3D [2], MICC Florence [30], and the NoW Challenge [6]. Table 17 summarizes representative parametric, generative, and direct geometry estimation-based 3D face reconstruction approaches, providing a structured basis for analyzing quantitative performance with standard evaluation metrics. Early parametric reconstruction methods such as 3DDFA [2] rely on sparse landmark supervision, providing stable global alignment but limited high-frequency detail. Subsequent dense correspondence and mesh-based approaches, including PRNet [8] and DF2Net [5], improve geometric accuracy through vertex-level supervision. More recent geometric estimation-based frameworks such as DECA [7], EMOCA [18], and Faceverse [121] incorporate expression-aware modeling and refinement modules.

While Table 17 shows the reconstruction pipelines, recent research has increasingly explored neural rendering approaches that integrate facial priors with implicit scene representations. Rendering-oriented approaches optimize image synthesis objectives rather than explicit mesh reconstruction. As discussed earlier, their evaluation commonly relies on perceptual image similarity metrics such as PSNR, SSIM [124], and LPIPS [26]. Table 18 lists face-specific reconstruction systems that integrate implicit or neural rendering representations.

Recent work has further emphasized the importance of fair and modular evaluation protocols. For example, Sariyanidi et al. [141] proposed a benchmark framework that decomposes 3D face reconstruction error into interpretable components, enabling more granular and transparent comparison across methods and datasets. As discussed earlier, rendering-based methods such as NeRFs and Gaussian splatting represent scenes using implicit radiance fields or point-based primitives rather than explicit meshes. As a result, geometric error cannot always be computed directly [112]. Consequently, these methods are typically evaluated using perceptual metrics such as PSNR, SSIM [124], and LPIPS [26], which better capture visual fidelity than explicit geometric accuracy.

To clarify evaluation practices, Table 19 shows perceptual (left) and geometric (right) reporting standards within face reconstruction research. A fundamental distinction is made between rendering-oriented and geometry-oriented evaluation paradigms in 3D face reconstruction. Unlike PSNR and SSIM [124] where higher values indicate better perceptual quality, LPIPS [26] measures perceptual distance, and therefore lower values correspond to improved visual similarity. Similarly, geometry-based metrics represent reconstruction error, where lower values indicate better performance. Rendering-focused approaches optimize photometric realism, as reflected by higher PSNR and SSIM [124] scores on datasets such as FaceScape [33], where methods like SplatFace [137] and Mip-Splatting [142] outperform 3DGS Avatar [113] in visual fidelity. On in-the-wild data, models such as PointAvatar [136], NerFace [143], and IMAvatar [118] demonstrate comparatively strong SSIM values despite moderate PSNR. Additionally, MonoAvatar [134] and NeRFBlendshape [131] illustrate lower LPIPS [26], reflecting improved visual similarity despite moderate PSNR, suggesting that perceptual consistency can be maintained while generalization to unconstrained real-world conditions remains challenging due to domain discrepancies.

In contrast, geometry-oriented methods prioritize structural accuracy. On the NoW benchmark [6], TokenFace [144] and MICA [145] achieve lower scan-to-mesh errors than earlier methods such as FlowFace [146] and PRNet [8], which rely heavily on synthetic training data. This improvement highlights the benefit of more robust representation learning and improved identity modeling for real-world geometric reconstruction. Earlier parametric reconstruction-based approaches such as PRNet [8] and 3DDFA-V2 [40] exhibit higher reconstruction errors on datasets, including MICC [30] and AFLW2000 [2], while SynergyNet [12] and SADRNet [147] demonstrate incremental improvements in RMSE and NME, reflecting progressive refinement of parametric reconstruction.

**Table 19:** Benchmark of rendering-oriented and geometry-oriented evaluation paradigms in 3D face reconstruction. Rendering methods are evaluated using perceptual metrics, while geometry-oriented methods are evaluated using dataset-specific metrics.

Rendering-Oriented Methods					Geometry-Oriented Methods (↓)					
Method	PSNR ↑	SSIM ↑	LPIPS ↓	Dataset	Method	Median	Mean	Std	Dataset	Metric
3DGS Avatar [113]	25.34	0.8350	0.1404	FaceScape	MICA [145]	0.90	1.11	0.92	NoW	S-M
Mip-Splatting [142]	25.60	0.8387	0.1379	FaceScape	TokenFace [144]	**0.76**	**0.95**	**0.82**	NoW	S-M
SplatFace [137]	**26.58**	**0.8556**	**0.1193**	FaceScape	FlowFace [146]	0.87	1.07	0.88	NoW	S-M
PointAvatar [136]	21.12	0.874	0.117	i-t-w	PRNet [8]	1.50	1.98	1.88	NoW	S-M
NerFace [143]	22.7	**0.877**	0.126	i-t-w	3DDFA-V2 [40]	–	2.04	–	MICC	RMSE
MonoAvatar [134]	**22.77**	0.795	**0.100**	i-t-w	SynergyNet [12]	–	**1.87**	–	MICC	RMSE
IMAvatar [118]	22.4	0.874	0.178	i-t-w	3DDFA-V2 [40]	–	3.56	–	AFLW2000	NME
NeRFBlendshape [131]	22.77	0.793	0.110	i-t-w	SADRNet [147]	–	**3.46**	–	AFLW2000	NME

i-t-w: In-the-wild; S-M: Scan-to-mesh; NME: Normalized Mean Error (%); RMSE: Root Mean Square Error (mm); ↓ less is better; ↑ higher is better; **Bold values** indicate the best performance among the compared methods in the dataset.

**Figure 8:**
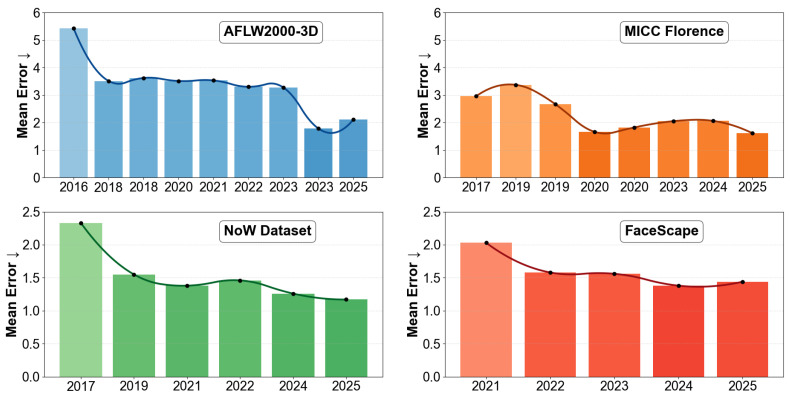
Performance trends of representative 3D face reconstruction methods across commonly used benchmarks. The plots show the evolution of reconstruction accuracy measured by mean error on four widely adopted datasets: AFLW2000-3D yearwise [2,8,40,96,130,148,149,150,151]; MICC Florence [30], yearwise [3,5,92,128,152,153,154,155]; NoW [6], yearwise [6,7,92,145,156,157]; and FaceScape [33], yearwise [7,121,144,158,159]. Bars represent reported quantitative results from representative state-of-the-art methods, while the smooth curves illustrate overall performance trends over time. ↓ less is better.

Both rendering and geometry-based methods exhibit performance degradation under domain shift conditions, with lower accuracy on in-the-wild datasets and the NoW benchmark [6] when trained on synthetic data. Nevertheless, year-over-year improvements indicate that recent methods are progressively narrowing this gap through more robust learning, hybrid supervision, and enhanced generalization strategies. These results are demonstrated in Figure 8, suggesting that improvements in reported accuracy are not solely attributable to architectural advances but are also influenced by dataset characteristics and evaluation protocols.

The quantitative results summarized in Figure 8 illustrate the evolution of reconstruction accuracy across widely used benchmarks, revealing a consistent reduction in reconstruction error over time. Early approaches such as 3DDFA [2] reported relatively high errors on **AFLW2000-3D**, indicating the limitations of early parameter regression methods that relied primarily on sparse landmark supervision. Subsequent models incorporating dense correspondence learning and improved deep learning architectures significantly reduced this error. For example, Pix2Vertex (2018) [96] and PRNet (2018) [8] introduced dense regression strategies that improved geometric reconstruction accuracy. Later works such as DSFNet (2023) [151] and FlowFace (2023) [148] further refined these architectures, leading to improved performance, with Pixel3DMM (2025) [130] achieving errors close to 2.11.

A similar trend can be observed for the **MICC Florence** dataset [30], where early reconstruction pipelines such as VRN (2017) [160] and DF2Net (2019) [5] reported errors around 2.96 and 3.37 respectively. With the introduction of improved supervision strategies and hybrid learning frameworks, reconstruction accuracy gradually improved. Recent methods, including RAFaRe (2023) [128], Hybrid Shape Deformation (2024) [152], and Hierarchical MLANET (2025) [154], demonstrate lower reconstruction errors, highlighting the benefits of improved geometric modeling and training strategies. For the **NoW** dataset [6], which is considered a challenging benchmark emphasizing high-precision geometric reconstruction, substantial improvements are also observed. Early methods such as 3DMM-CNN (2017) [92] reported errors of approximately 2.33, while subsequent models, including RingNet (2019) [6] and DECA (2021) [7], significantly improved reconstruction accuracy. More recent frameworks such as Hybrid-Level Context (2024) [156] and Hi3DFace (2025) [157] achieve errors approaching 1.17, indicating the increasing effectiveness of modern encoder–decoder architectures and improved facial representation models. The **FaceScape** dataset [33], which contains high-quality facial scans with detailed expression variations, also demonstrates similar performance improvements. Methods such as Convincing 3D [144] initially reported errors around 2.03, while more recent approaches, including Faceverse (2022) [121], TokenFace (2023) [144], Dynamic 3D [158], and WarpHE4D (2025) [159], have further reduced reconstruction errors to approximately 1.4.

The results highlight three key trends in 3D face reconstruction. Dense geometry regression and improved representations have substantially reduced reconstruction error compared to early landmark-based methods. Hybrid strategies that combine parametric models with deep networks enhance cross-dataset generalization. Finally, recent work emphasizes geometric fidelity and expression modeling, driving steady gains across benchmark datasets.

**Figure 9:**
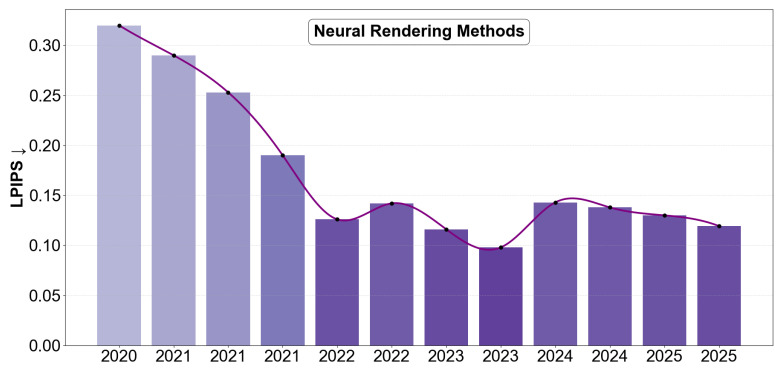
Temporal evolution of perceptual reconstruction quality in neural rendering-based facial modeling methods. LPIPS [26] scores reported in the literature are aggregated across representative approaches from 2020 to 2025 [110,137,142,161,162,163,164,165,166,167,168,169] respectively. Lower LPIPS values indicate improved perceptual similarity between rendered outputs and reference images. ↓ less is better.

Figure 9 highlights the evolution of neural rendering facial modeling methods evaluated using the LPIPS [26] perceptual similarity metric, where lower values indicate improved perceptual reconstruction quality. LPIPS was chosen as it correlates much better with human perceptual judgment, especially for neural rendering and photorealistic face reconstruction tasks [26]. Early neural rendering approaches such as NeRFs (2020) [110] reported relatively high LPIPS values around 0.32, reflecting the limitations of early volumetric neural radiance field models when applied to detailed facial reconstruction tasks. Subsequent improvements introduced temporal modeling and deformation-aware representations, as demonstrated by NeRFies (2021) [161] and HyperNeRF (2021) [162], which reduced perceptual error to 0.29 and 0.253, respectively. These methods incorporated dynamic scene representations and improved deformation modeling, enabling better reconstruction of non-rigid facial motion. Further advancements were achieved with architectures that incorporated explicit facial priors and hybrid learning strategies. For instance, PixelNeRF (2021) [163] significantly reduced LPIPS to approximately 0.19, demonstrating the benefits of conditioning neural radiance fields on image-based feature embeddings. Later methods such as Fenerf (2022) [164] and Mofanerf (2022) [165] further improved perceptual reconstruction quality by integrating facial identity modeling and expression-aware representations, achieving LPIPS values around 0.126–0.142.

Recent work has focused on improving rendering efficiency and geometric representation through point-based and Gaussian-based neural rendering frameworks, resulting in a clear reduction in perceptual error, particularly evident around 2023. Methods such as Pointavatar (2023) [136] and Gaussian3Diff (2023) [167] achieved additional improvements, reducing LPIPS to 0.098. These approaches leverage explicit geometric primitives and efficient rendering pipelines, enabling higher perceptual fidelity while maintaining computational efficiency. Methods such as Mip-Splatting (2024) [142] and GaussianAvatars (2024) [166] demonstrate competitive LPIPS performance, achieving values of approximately 0.1379 and 0.143, respectively. However, a temporary increase in perceptual error may be observed, potentially reflecting a trade-off where stronger geometric constraints such as explicit shape regularization or alignment supervision prioritize structural consistency at the expense of photometric optimization, while Avat3r (2025) [169] and SplatFace (2025) [137] further improve perceptual reconstruction quality, achieving LPIPS [26] values close to 0.13 and 0.1193. These results highlight the growing impact of gaussian splatting-based representations and hybrid neural rendering pipelines, which offer improved scalability and perceptual realism compared to earlier volumetric methods. The reported values are compiled from face-specific reconstruction settings across heterogeneous datasets and, while often evaluated under similar experimental conditions, should be interpreted as indicative trends rather than directly comparable results. Variations in datasets, evaluation protocols, alignment procedures, and acquisition conditions may influence the reported performance.

Overall, the trend indicates a steady reduction in perceptual reconstruction error over time, reflecting significant progress in neural rendering techniques. Early volumetric radiance-field models primarily emphasized view synthesis quality, whereas more recent point-based rendering methods achieve lower perceptual error due to improved modeling of fine appearance details and efficient rendering. Subsequent approaches increasingly incorporate facial priors, deformation models, and geometry-aware constraints to improve structural consistency and identity preservation. While these enhancements improve geometric stability, they may introduce trade-offs with purely perceptual optimization, which can partially explain the relatively higher LPIPS values [26] observed in some later methods.

### 3.2. Interpretation of Benchmark Results

The benchmark results presented in Section 3.1 reveal broader trends in modern deep learning-based 3D face reconstruction. While advances in network design and supervision contribute to performance gains, the reported results are also strongly influenced by dataset properties, sensing modalities, and evaluation protocols. Benchmark comparisons should therefore be interpreted in the context of both reconstruction paradigms and data acquisition conditions.

**The impact of sensing modalities and dataset construction** directly affects the reliability of reconstruction accuracy. Datasets acquired using high-precision structured-light scanners or controlled multi-view capture systems typically provide dense and accurate ground-truth geometry, generally leading to lower reconstruction error during evaluation. In contrast, datasets based on monocular RGB imagery or consumer-grade depth sensors often contain measurement noise, calibration inaccuracies, and illumination variability that increase reconstruction difficulty. Multi-view capture further improves geometric consistency by reducing occlusion ambiguity and strengthening triangulation accuracy. As a result, differences in benchmark performance may partly reflect sensor fidelity and acquisition quality rather than purely algorithmic progress.

**The synthetic to real domain performance gap** is a recurring observation across benchmark evaluations. Models trained on synthetic datasets often achieve high reconstruction accuracy due to controlled rendering conditions and dense annotations; however, they may exhibit reduced generalization when applied to in-the-wild imagery. This discrepancy arises because synthetic data fails to fully capture real-world variability, including complex lighting effects, partial occlusions (e.g., object interactions), and demographic diversity across age, ethnicity, and facial morphology.

**Architectural trends in reconstruction models** have substantially improved reconstruction accuracy over the past decade. Encoder–decoder models combined with parametric face priors typically provide strong global alignment and stable identity preservation. Methods based on dense correspondence modeling, graph-based mesh refinement, and differentiable rendering further improve geometric fidelity by enforcing structural constraints during optimization. More recently, neural rendering approaches based on implicit radiance fields or point-based representations have achieved strong perceptual quality, although this often introduces trade-offs between photorealistic rendering and explicit geometric accuracy.

**The trade-off between reconstruction fidelity and efficiency** highlights a clear balance between reconstruction accuracy and computational cost. High-fidelity methods often rely on refinement modules, graph-based operations, or adversarial supervision, increasing computational complexity and hardware requirements. In contrast, lightweight parametric approaches emphasize efficiency and scalability, enabling near real-time inference at the expense of fine-scale detail. This observation is further supported by supervision strategies: sparse landmark supervision provides robust global alignment but limits surface detail, whereas dense vertex-level supervision improves geometric accuracy but depends on expensive 3D ground-truth acquisition.

Overall, these findings suggest a fundamental trade-off between reconstruction accuracy and computational efficiency. Higher accuracy is typically achieved through increased model complexity, iterative optimization, or geometric refinement, which reduces inference speed. In contrast, lightweight regression-based models enable real-time performance but may sacrifice fine geometric detail. Notably, recent architectures discussed above demonstrate that efficient network design can partially mitigate this trade-off, enabling both low reconstruction error and high inference speed.

### 3.3. Limitations of the Survey

While this survey provides a structured overview of deep learning-based 3D face reconstruction, several limitations should be acknowledged. First, the discussion primarily focuses on reconstruction pipelines that estimate 3D facial geometry from monocular RGB imagery as these represent the most widely studied and practically deployable configurations in current research. Consequently, multi-view studio capture systems and specialized scanning setups are discussed mainly in the context of dataset acquisition rather than detailed rendering-oriented system design, which may underrepresent research on photorealistic avatar synthesis and view-consistent rendering environments. Second, quantitative benchmarking is constrained by heterogeneity across evaluation datasets. Existing benchmarks differ in capture conditions, sensing modalities, subject diversity, annotation protocols, and metric definitions, which complicates direct cross-dataset comparison. Although metrics are aligned where possible, these inconsistencies limit strict quantitative comparability between the reported results. Third, the rapid evolution of neural rendering and generative modeling continues to introduce new hybrid architectures that may not yet be fully represented in standardized benchmarks. As a result, some recent high-fidelity approaches currently lack extensive longitudinal evaluation. Fourth, proprietary systems and large-scale industry datasets are often not publicly accessible. Such systems may employ additional sensing configurations, calibration pipelines, and optimization strategies that cannot be fully examined within the scope of this survey. Finally, this survey primarily emphasizes methodological and system-level analysis. Broader considerations, including ethical implications, demographic fairness, privacy protection, and regulatory compliance, are outside the present scope but represent important directions for future research. These limitations do not diminish the contributions of this survey; rather, they clarify its focus on reproducible, sensing-aware, and deployment-oriented 3D face reconstruction pipelines.

## 4. Conclusions and Future Directions

This survey reviewed the evolution of deep learning-based 3D face reconstruction through a structured sensing-to-inference pipeline. By synthesizing advances in parametric modeling, differentiable rendering, dense correspondence learning, and neural refinement strategies, we demonstrate that reconstruction performance is governed not only by model architecture but also by the interaction between sensing conditions and evaluation protocols. Our analysis highlights that 3D face reconstruction should be treated as a *system-level problem*, where benchmark results cannot be interpreted independently of acquisition conditions, camera modeling assumptions, landmark configurations, or alignment strategies. Reported performance gains therefore reflect coordinated improvements across sensing, representation, and evaluation components rather than architectural design alone. This perspective underscores the need for transparent reporting standards and reproducible benchmarking practices.

Despite substantial progress, important challenges remain. Current approaches must balance geometric fidelity, perceptual realism, and computational efficiency. High-fidelity reconstruction often relies on dense supervision, refinement modules, or adversarial training, increasing computational cost and data acquisition complexity. A key challenge in 3D face reconstruction lies in balancing geometric fidelity with data scalability. Densely supervised approaches based on high-quality 3D scan datasets provide strong geometric constraints and improved reconstruction accuracy; however, their scalability is inherently limited due to the high cost and controlled environments required for data acquisition. Hybrid and self-supervised approaches provide a promising direction by combining limited 3D annotations with large-scale 2D data to balance accuracy and generalization. Lightweight parametric models enable real-time inference but may sacrifice fine-scale geometric detail, highlighting a persistent trade-off between efficiency and reconstruction fidelity. Future research should emphasize unified evaluation protocols that jointly consider geometric accuracy and perceptual realism in standardized alignment procedures. Benchmarking should also document hardware assumptions, runtime characteristics, and calibration configurations to enable fair comparison. Improving cross-dataset generalization and robustness under real-world conditions remains critical, particularly in addressing domain gaps between synthetic and real data. Overall, this survey provides a structured and practically grounded foundation for developing robust, interpretable, and deployment-ready 3D face reconstruction systems.

## Figures and Tables

**Table 1:** Comparison of existing surveys on Three-Dimensional (3D) face reconstruction across general coverage dimensions.

Survey	Methodology	Datasets and Acquisition	Sensor/Calibration	Algorithm
Zollhöfer et al. (2018) [22]	✓	✓	×	×
Kammoun et al. (2020) [20]	✓	×	×	×
Sharma and Kumar (2022) [19]	✓	✓	×	×
Guo et al. (2023) [21]	✓	✓	×	✓
Diao et al. (2024) [24]	✓	✓	×	✓
Ours	✓	✓	✓	✓

✓: covered; ×: not covered.

**Table 2:** Comparison of recent 3D face reconstruction surveys highlighting key differences in sensing awareness, evaluation strategies, and system-level analysis.

Comparison Dimension	Sharma & Kumar (2022) [19]	Guo et al. (2023) [21]	Diao et al. (2024) [24]	Ours
Sensing Hardware Analysis	×	×	×	✓
Synthetic-to-Real Generalization	×	×	×	✓
Unified End-to-End Pipeline	×	✓	×	✓
Implementation Framework	×	×	×	✓
Benchmark Trend Analysis	✓	✓	✓	✓
Neural Rendering Coverage	×	✓	×	✓

✓: covered; ×: not covered.

**Table 3:** Public datasets used for 3D face reconstruction. For depth-based datasets, the numbers denote the number of subjects. For landmark-based datasets, the numbers indicate annotated images or video frames.

Reference	Dataset	Samples	Annotation Type	Description
**Depth-Based Datasets**				
[27]	Basel Face Model (BFM)	200 scans	3D scans	3D morphable model
[15]	FaceWarehouse	150 subjects	3D meshes	Expression dataset
[30]	MICC Florence	53 subjects	3D scans	Evaluation benchmark
[31]	BU-3DFE	100 subjects (≈2.5K scans)	3D scans	Expression database
[32]	4DFAB	180 subjects	4D sequences	Dynamic facial dataset
[16]	LSFM	9663 subjects	3D scans	Large-scale 3DMM
[33]	FaceScape	359 subjects	High-quality 3D scans	Large-scale 3D dataset
[6]	NoW Challenge	100 subjects	3D scans	Reconstruction-based
**Multi-View/Video-Based Datasets**				
[34]	ZJU-MoCap	9 subjects	Multi-view videos	Dynamic human
[35]	VoxCeleb	1M+ utterances	Video frames	In-the-wild heads
[36]	300-VW-3D	114 videos	68 landmarks	Video-based
**Landmark-Based Datasets**				
[37]	300W-LP	61,225 images	68 landmarks	In-the-wild dataset
[29]	CelebA	202,599 images	5 landmarks	Attribute dataset
[38]	3DFAW	≈23K frames	66 landmarks	Face alignment
[2]	AFLW2000-3D	2000 images	68 landmarks	Evaluation-based
[39]	AFLW-LFPA	≈1.3K images	21 landmarks	Large-pose alignment

**Table 4:** Comparison of dataset categories used in 3D face reconstruction research.

Property	3D Scan-Based	In-the-Wild Image	Synthetic	Dynamic (4D)
**Primary Data Type**	3D meshes	RGB images (2D)	Rendered images with ground-truth geometry	Temporal 3D sequences
**Ground Truth Geometry**	Dense mesh	Weak or indirect supervision	Ground-truth geometry	Dense dynamic meshes
**Capture Setup**	Structured-light scanners or multi-view capture rigs	Single RGB camera or internet image collections	Rendering pipeline using parametric models	Multi-camera dynamic capture systems
**Acquisition Cost**	High	Low	Moderate	Very high
**Dataset Scale**	Small–medium	Very large	Large (synthetic generation)	Small–medium
**Representative Datasets**	BU-3DFE [31], FaceWarehouse [15]	AFLW [39], CelebA [29], 300W-LP [37]	Rendered 3DMM datasets [2,43]	BU-4DFE [42], D3DFACS [41]
**Typical Use**	Dense supervision	Sparsely supervised learning	Pretraining or data augmentation	Temporal reconstruction and expression tracking

**Table 5:** Mapping between sensing modalities and their influence on 3D face reconstruction performance. The table summarizes how different acquisition sensors provide distinct measurement characteristics and introduce trade-offs in geometric fidelity, robustness, and reconstruction reliability.

Sensor Type	Primary Measurement Characteristic	Impact on Performance
RGB Camera	High-resolution texture without explicit depth	Strong texture guidance; missing depth increases geometric ambiguity [8]
Structured Light	Dense active depth with high local precision	High geometric accuracy under controlled lighting; performance degrades with reflective materials [49]
Time-of-Flight (ToF)	Real-time depth sensing with moderate accuracy	Robust depth capture; reduced fine-detail accuracy [50]
Multi-View RGB Systems	Triangulation from synchronized calibrated views	Improved occlusion handling and mesh stability, often reducing geometric reconstruction error [51]
LiDAR Sensors	Accurate global depth with sparse sampling	Strong global geometry; limited fine surface detail [52]

**Table 12:** Summary of landmark and correspondence configurations used in representative 3D face reconstruction studies. Sparse configurations typically employ ≤100 semantically defined keypoints, whereas dense configurations rely on extended landmark sets or mesh-based vertex correspondences as reported in the respective publications.

Reference	Training Data	Evaluation Data	Landmark/Correspondence	Type
[14]	Synthetic data	NoW, MICC	Mesh-based supervision	Dense
[12]	300W-LP	AFLW2000-3D, MICC	68 landmarks	Sparse
[93]	Custom multi-view	3DFAW	Sparse landmark constraints	Sparse
[94]	300W-LP	AFLW2000-3D, MICC	68 landmarks	Sparse
[5]	Custom dataset	MICC Florence	Landmark + mesh supervision	Dense
[95]	Generated 3D renders	AFLW2000-3D, AFLW-LFPA	Mesh-based correspondence	Dense
[32]	4DFAB	MICC Florence	Sparse landmark constraints	Sparse
[3]	300W-LP	AFLW2000-3D	68 landmark constraints	Sparse
[96]	Custom dataset	MICC Florence	Mesh vertex supervision	Dense
[97]	Pre-trained (BFM, 3DDFA)	MICC Florence	Mesh vertex supervision	Dense

**Table 13:** Loss functions employed by state-of-the-art 3D face reconstruction methods.

Method	# Obj.	# Landmarks	Photo.	Perc.	Shape/Expr.	Vertex	Adv.	Geom. Supervis
3DDFA [2]	3	68	No	No	2	No	No	Sparse
PRNet [8]	1	–	No	No	No	Dense	No	Dense
GANFit [11]	6	68	Yes	Yes	2	No	Yes	Sparse + texture
DF2Net [5]	4	68	No	No	2	Yes	No	Hybrid
SynergyNet [12]	3	68	No	No	2	No	No	Sparse
DECA [7]	5	68	Yes	Yes	2	No	No	Sparse + appear.
EMOCA [18]	5	68	Yes	Yes	2	No	No	Sparse + appear.
RingNet [6]	3	68	No	No	2	No	No	Sparse
Faceverse [121]	5	68	Yes	Yes	2	No	No	Sparse + appear.
Pix2Vertex [96]	1	–	No	No	No	Dense	No	Dense
Deep3DFaceRecon [3]	4	68	Yes	No	2	No	No	Sparse + appear.
HRN [122]	5	68	Yes	Yes	2	No	No	Sparse + appear.

# Obj. = number of distinct loss families employed; # Landmark = number of 2D keypoints; Photo. = photometric consistency loss; Perc. = perceptual/identity loss; Shape/Expr. = parametric regularization terms; Vertex = dense mesh supervision; Adv. = adversarial loss; Geom. Supervis = geometric supervision type; appear. = appearance.

**Table 14:** The table categorizes metrics according to their evaluation objective and highlights their dependence on sensing hardware and acquisition modalities.

Metric Category	Metrics	Measures	Hardware Dependency	Preferred Sensors
Geometric Accuracy	NME, RMSE, scan-to-mesh	Deviation from ground-truth geometry; and are influenced by alignment procedures and mesh resolution.	High	Structured light scanners, multi-view RGB, high-precision depth
Perceptual Quality	PSNR, SSIM, LPIPS	Visual realism and texture fidelity rather than geometric accuracy.	Medium	High-resolution RGB cameras, controlled lighting
Landmark Alignment	2D landmark error, reprojection error	Consistency between projected 3D landmarks and annotated keypoints.	Medium	High-resolution RGB, multi-view rigs
Computational Efficiency	FPS, runtime, memory	Real-time capability and deployment feasibility.	High	RTX GPUs, edge GPUs, optimized embedded systems

**Table 15:** Quantitative comparison of 3D face reconstruction performance across sensing modalities. Values are aggregated from representative studies and normalized for comparison. Geometric accuracy is reported using RMSE or NME as defined in the respective benchmarks, and FPS indicates approximate inference speed on GPU-based systems. Lower error indicates better reconstruction accuracy, while higher FPS indicates improved efficiency.

Sensor Type	Dataset	Setup	RMSE ↓	NME ↓	FPS ↑
Monocular RGB	AFLW2000-3D [2], NoW [6]	Single RGB camera	3.0–4.0 mm	2.0–3.5	∼25–30 [3,8]
ToF	MICC Florence [30]	Kinect v2/ds	2.0–3.0 mm	1.3–2.0	∼20–25 [22,40]
Structured Light	FaceWarehouse [15], BU-3DFE [31]	3dMD/s-l scanner	0.5–2.0 mm	1.0–1.5	∼10–15 [22]
Multi-view RGB	NoW Challenge [6], ZJU-MoCap [34]	Multi-camera rig	1.0–2.0 mm	0.8–1.2	∼8–15 [10]
Mobile LiDAR	ARKit [126]	iPhone LiDAR	4.0–10.0 mm	1.5–3.0	∼15–20 [57]

s-l: structured light; ds: depth scanner; ToF: time-of-flight; ↓: less is better; ↑: higher is better.

**Table 16:** Computational characteristics of representative 3D face reconstruction methods. Runtime categories are normalized from the respective publications, where real-time corresponds to approximately >25 FPS, near real-time corresponds to approximately 10–25 FPS, and offline indicates slower iterative optimization or batch processing.

Method	CPU Inference	Runtime/Image	Approx. FPS	(NME %) ↓	Reconstruction Paradigm
3DDFA * [2]	Yes	39.17 ms	25.5	5.4	CNN-based parameter regression
PRNet ^+^ [8]	Yes	9.8 ms	102	3.6	Dense position map regression
GANFit [11]	No	2000 ms	0.3–0.6	2.0	GAN-based optimization
DF2Net ^+^ [5]	No	500–1000 ms	1–2	3.5	Dense fitting with refinement
SynergyNet * [12]	Yes	0.33 ms	3000	3.3	Single-pass regression
DECA * [7]	Yes	8.3 ms	120	3.5	Parametric + differentiable
EMOCA * [18]	No	20–30 ms	33–50	3.0	Expression-aware extension
RingNet * [6]	Yes	15–25 ms	40–65	1.8	FLAME-based regression
Faceverse * [121]	Yes	33.3 ms	30	2.5	Parametric modeling
Pix2Vertex ^+^ [96]	No	4.79 ms	209	4.0	Dense vertex regression
MGCNet ^+^ [127]	No	300–800 ms	3	2.5	Mesh-based graph refinement
Deep3DFaceRecon * [3]	Yes	10–20 ms	100	2.8	Efficient CNN regression
HRN * [122]	Yes	5–12 ms	180	2.6	Lightweight CNN regression
3DMM fitting * [4]	Yes	2000–5000 ms	0.2–0.5	8.0	Iterative 3DMM fitting

CPU Inference indicates whether the method can be executed without GPU acceleration as reported in the original publication. Computational burden reflects relative model complexity and optimization cost described by the respective methods. NME Normalized Mean Error; * Parametric reconstruction-based methods; ^+^ Direct geometry estimation; ↓: less is better.

**Table 17:** 3D face reconstruction methods evaluated on standard facial benchmarks. This table summarizes representative geometry-oriented 3D face reconstruction approaches, including parametric regression, mesh-based estimation, and hybrid GAN–geometry modeling pipelines.

Method	Year	Representation	Landmarks	Training Dataset	Evaluation Dataset
3DDFA [2]	2016	3DMM	68	300W-LP	AFLW2000-3D
PRNet [8]	2018	Dense Mesh	Dense	Synthetic	MICC Florence
DF2Net [5]	2019	GCN Mesh	68	4DFAB	MICC Florence
RingNet [6]	2019	FLAME	Sparse	Synthetic	NoW Challenge
GANFit [11]	2019	3DMM + GAN	Sparse	Synthetic	AFLW2000-3D
Deep3DFaceRecon [3]	2020	3DMM	Sparse	Synthetic + VGGFace2	AFLW2000-3D
MGCNet [127]	2020	Mesh CNN	Dense	FaceWarehouse	MICC Florence
3DDFA-V2 [40]	2020	3DMM	68	300W-LP	AFLW2000-3D
SynergyNet [12]	2021	3DMM + CNN	68	300W-LP	AFLW2000-3D
DECA [7]	2021	FLAME	Sparse	Synthetic	NoW Challenge
Faceverse [121]	2022	Parametric	Sparse	Synthetic + Multi-View	NoW Challenge
RAFaRe [128]	2023	Non-parametric	Dense	Synthetic + Pseudo-2D/3D	AFLW2000-3D/MICC
Advanced3DFace [129]	2024	Mesh + GAN + GNN	Sparse	Synthetic + Multi-View	AFLW2000-3D/NoW
Pixel3DMM [130]	2025	Parametric + Dense	Dense	Synthetic + In-the-Wild	AFLW2000-3D/NoW

**Table 18:** Representative face-specific neural rendering reconstruction methods. These approaches integrate facial priors or implicit representations to enhance photometric realism while modeling 3D facial structure. MV = Multi-View, Mono. = Monocular.

Method	Year	Representation	Training Dataset	Evaluation Dataset	Facial Prior
NeRFBlendshape [131]	2021	Blendshape-NeRF	MV Capture	Synth./Real	Blendshape
HeadNeRF [132]	2022	NeRF	MV Capture	Internal	3DMM
IMAvatar [118]	2022	Implicit RF	Mono. Video	Internal	FLAME
NHA [133]	2022	Vol. Field	MV Capture	Internal	Parametric
MonoAvatar [134]	2022	Implicit Field	Mono. Video	Internal	Mesh anchor
INSTANT [135]	2023	Volumetric Neural Field	Mono. Video	Captured	FLAME
PointAvatar [136]	2023	Point-NeRF	MV Capture	Internal	3DMM fit
SplatFace [137]	2023	3DMM-GS	FaceScape	FaceScape	3DMM
3DGS Avatar [113]	2023	3DGS	MV Capture	Internal	Param. initial
FlashAvatar [138]	2024	3D Gaussian Splatting	Mono. Video	Captured	FLAME
NeRFlame [139]	2024	FLAME-NeRF	MV Synthetic	FaceScape	FLAME
HeadAvatar [140]	2025	Deformable Implicit Field	Mono. Video	In-the-wild	Deform prior

MV: Multi-View; Mono.: Monocular; RF: radiance field; GS: Gaussian splatting. Internal: Non-public datasets; Captured: Custom real-world data; Synth./Real: Synthetic and real data.

## Data Availability

The original contributions presented in this study are included in the article. Further inquiries can be directed to the corresponding author.
